# Distinct cell classes in the superior paraolivary nucleus (SPN) region in the gerbil auditory brainstem revealed by *in vivo* physiological and anatomical characterization

**DOI:** 10.1016/j.heares.2025.109202

**Published:** 2025-01-27

**Authors:** T.P. Franken, P.X. Joris, P.H. Smith

**Affiliations:** a Laboratory of Auditory Neurophysiology, University of Leuven, Herestraat 49 bus 1021, B-3000, Leuven, Belgium; b Department of Neuroscience, University of Wisconsin School of Medicine and Public Health, 1111 Highland Avenue, Room 5505 WIMR-II, Madison, WI, 53705, USA; c Department of Neuroscience, Washington University School of Medicine, St Louis, MO, USA

**Keywords:** Superior olivary complex, Hearing, Brainstem, Rebound, Binaural, Temporal, Off-responses

## Abstract

The superior para-olivary nucleus (SPN or SPON) is a prominent nucleus in the superior olivary complex of the auditory brainstem. The cellular composition of the nucleus reportedly differs between species, but a prominent recurring feature is the unusual characteristic to not respond during a sound but at its offset. Blocking glycine has shown that sound-induced inhibition is the mechanism, but the time course of the responsible synaptic events has not been directly measured *in vivo*. We obtained intracellular recordings in the Mongolian gerbil (*meriones unguiculatus*) with patch electrodes containing biocytin, and retrieved 12 labeled neurons with large dendritic trees within and around the SPN region. We found that these neurons could be categorized into three classes that show consistency along multiple dimensions like ultrastructure, spontaneous activity, and responses to current injection and a variety of ipsi- and contralateral sounds. Fast cells fire at onset of depolarizing current, generate short-latency rebound spikes to sound or hyperpolarizing current, and show dense synaptic coverage. Slow cells show sparse synaptic coverage, sustained responses to depolarization, and inhibition with a slow time course to hyperpolarizing current or sound. Uninhibited cells form a third class which profoundly differ in their responses to sound, lacking rebound spiking. We propose that fast cells project to the inferior colliculus, and slow cells to the cochlear nucleus.

## 1. Introduction

In the auditory brainstem of humans (Kulesza and Grothe, 2015) and other species, one of the prominent periolivary nuclei is the superior paraolivary nucleus (SPN or SPON) also referred to in non-rodent species as the dorsomedial periolivary nucleus (DMPO) (Morest, 1968; Spangler et al., 1987; Warr, 1972). SPN is situated medial to the lateral superior olive (LSO) and dorsal to the medial superior olive (MSO) and medial nucleus of the trapezoid body (MNTB). Based on extracellular response features *in vivo*, SPN neurons have been implicated in several functions of auditory processing including enhancing monaural signal-to-noise ratios, sound localization (Dehmel et al., 2002), duration coding (Kadner et al., 2006; Kulesza et al., 2007), gap detection (Kadner and Berrebi, 2008), encoding sound rhythms (Felix et al., 2011) and forward masking (Gao et al., 2017).

The cellular composition of this nucleus may vary depending on the species. In rats and mice, there seems to be a single predominant anatomical population. Here, the vast majority of SPN cells have round cell bodies, multipolar dendritic trees, are GABAergic (Helfert et al., 1989; Kulesza and Berrebi, 2000) and send their inhibitory projection by way of the lateral lemniscus primarily to the ipsilateral central nucleus and dorsal cortex (DC) of the inferior colliculus (IC) (Coleman and Clerici, 1987; Saldanã et al., 2009). In contrast, the SPN of gerbils and guinea pigs may contain multiple cell types. Anatomically, 3 cell shapes were initially identified in gerbil: round, triangular and spindle (Nordeen et al., 1983). Subsequent studies in guinea pig (Schofield, 1995, 1991; Schofield and Cant, 1992, 1991) indicated that large multipolar cells with round cell bodies project primarily to the IC bilaterally but mostly to the ipsilateral IC while elongated cells project to the cochlear nucleus (CN), predominantly ipsilaterally, and some smaller cells project to both IC and CN. Some of the CN-projecting cells immuno-labeled with GABA or glycine (Ostapoff et al., 1997). Guinea pig SPN neurons can also project to the superior colliculus (Mellott et al., 2018).

Inputs to SPN cells originate directly and indirectly from the contralateral CN with a smaller input arising directly from ipsilateral CN (Friauf and Ostwald, 1988; Kuwabara et al., 1991; Schofield, 1995; Thompson and Thompson, 1991a). One direct source arises from large axons in the intermediate acoustic stria that belong to glutamatergic octopus cells that form large boutons in SPN. A second source is from thinner multipolar cell axons that form smaller swellings presumed to be synaptic boutons. It is assumed that these axons arise from glutamatergic T-stellate cells, but this has not been confirmed (see Magnusson and Gomez-Alvarez, 2019). A major SPN input source is from the homolateral, glycinergic MNTB principal cells that are driven by contralateral globular bushy cell inputs in the form of the giant calyx of Held synapse (Banks and Smith, 1992; Helfert et al., 1989; Sommer et al., 1993). In mouse an average of 4 MNTB cells converge on a single SPN cell (Rajaram et al., 2020). Finally, there have been reports of MSO principal cell axons giving off excitatory collaterals to SPN (Kuwabara and Zook, 1999) as well as local presumably GABAergic collaterals from other SPN cells (Kulesza Jr. et al., 2000; Kulesza and Berrebi, 2000; Saldanã and Berrebi, 2000). In cat, labeling of physiologically identified neurons via axonal tracer injection showed inputs to DMPO from several classes of projection neuron in the contralateral CN; globular bushy cells (Smith et al., 1991), octopus cells (Smith et al., 2005), and multipolar cells (Smith et al., 1993), as well as from homolateral MNTB principal cells (Smith et al., 1998). The input from globular bushy cells seems to be absent in rodents and is regarded as the main difference between their SPN and the analogous structure, DMPO, in cat (Schofield, 1995).

As with the anatomical cell types, the *in vivo* extracellular response features of SPN cells are quite consistent in rats and mice but more variable in gerbils and guinea pigs. Extracellular recordings of spike activity from rats and mice (Felix et al., 2012, 2011; Kadner et al., 2006; Kopp-Scheinpflug et al., 2011; Kulesza et al., 2003) usually showed little effect from ipsilateral stimuli but this refers to suprathreshold not subthreshold activity. In contrast, most were inhibited by contralateral stimuli. This spike suppression during contralateral tone (or noise) presentations was occasionally preceded by an onset spike and frequently followed by an offset spike(s) response as the tone ended, regardless of tone duration. Blockade of glycine receptors *in vivo* eliminated this offset response (Kulesza, 2007) indicating that the offset spiking might be due to rebound from inhibition. Response features in the gerbil appear to be more heterogeneous (Behrend et al., 2002; Dehmel et al., 2002). Just over 50 % of the units were inhibited by contralateral inputs but only about half of these showed rebound firing. The remaining cells were excited by contralateral inputs typically showing onset, chopper or primary-like responses to short tones. In addition, cell firing was also affected by ipsilateral inputs for a sizeable percentage of these neurons. SPN cells also respond well to sinusoidally amplitude modulated (SAM) stimuli (Felix et al., 2012, 2011; Kulesza et al., 2003; Kuwada and Batra, 1999): cells with off-responses could phase-lock to modulation frequencies up to several hundred Hz again presumably due to the inhibitory rebound firing. In the cat DMPO, both onset and sustained chopper as well as sustained inhibition and offset responses were reported in response to tones (Guinan et al., 1972b, a).

*In vitro* recordings from mouse and gerbil SPN have helped to distinguish intrinsic and synaptic features that may act in shaping *in vivo* SPN cell responses (Felix II et al., 2017; Felix et al., 2013, 2011; Felix and Magnusson, 2016; Gómez-Álvarez et al., 2018; Kopp-Scheinpflug et al., 2015, 2011; Leijon et al., 2016; Yassin et al., 2014). Almost all SPN neurons responded to a hyperpolarizing current with very prominent rebound spiking at current offset that appears to be due largely to activation of a hyperpolarization-activated cation current (IH) and a T-type calcium conductance (ITCa). Shock stimulation of the MNTB elicits large glycinergic IPSPs in SPN neurons, made large in part by a very negative chloride reversal potential, that when terminated could also elicit offset spiking similar to that seen *in vivo* in response to contralateral tones. Blockade of GABA and glycine inhibition during IAS stimulation revealed convergence of only a few large fast glutamatergic inputs presumably from octopus cells. When stimulated together with the MNTB inputs, these octopus cell excitatory inputs could speed up the latencies of the MNTB-generated offset response. This change was NMDA mediated (Rajaram et al., 2019). The firing patterns of the spike responses to depolarizing current varied with some (48 %) showing onset responses, others (39 %) adapting responses and still others (13 %) burst responses. Anatomically the cell body size was similar in all response classes but the burst neurons tended to be situated more dorsolaterally, had more elongate cell bodies and longer and more complex dendritic trees (Felix et al., 2013).

No *in vivo* intracellular recordings are available for any SPN cells from any species. To begin to elucidate the *in vivo* intracellular response features of identified SPN neurons we have recorded from and labeled neurons in and around the gerbil SPN using patch electrodes.

## 2. Methods

The methods for surgery, *in vivo* patch clamp recording and light and electron microscopic histology have been previously described (Franken et al., 2018, 2016) and are briefly reviewed here. All procedures were approved by the KU Leuven Ethics Committee for Animal Experiments.

### 2.1. Animals and surgery

Mongolian gerbils (*Meriones unguiculates*) of either sex (P28-P36) were used. The animals were anesthetized by intraperitoneal injection of ketamine (80–120 mg/kg) and xylazine (8–10 mg/kg) in NaCl 0.9 %. Anesthetic maintenance was ensured by additional intramuscular injections of ketamine (30–60 mg/kg) and diazepam (0.8–1.5 mg/kg), following a positive toe pinch reflex. Body temperature was maintained at 37 ^◦^C with a homeothermic blanket (Harvard Apparatus, Holliston, MA, USA) and a heating lamp positioned above the animal. Exposure of the ventrolateral brainstem was done via a transbullar craniotomy. The pinnas were removed around the external acoustic meatus and the contralateral bulla opened as well to maintain acoustic symmetry. Meningeal layers overlying the exposed brainstem were removed prior to electrode penetration.

### 2.2. Electrophysiology

For intracellular recordings, patch clamp recordings were made with 5–7 MΩ pipettes pulled from borosilicate capillaries (1B120F-4, World Precision Instruments, Inc., Sarasota, FL, USA) with a horizontal puller (Model P-87, Sutter Instrument Co., Novato, CA, USA). The internal solution contained (in mM) 115 K gluconate (Sigma); 4.42 KCl (Fisher); 10 Na2 phosphocreatine (Sigma); 10 HEPES (Sigma); 0.5 EGTA (Sigma); 4 Mg-ATP (Sigma); 0.3 Na-GTP (Sigma); and 0.1–0.2 % biocytin (Invitrogen). pH was set at 7.3 using KOH (Sigma) and osmolality at 300 mmol/kg with sucrose (Sigma). A patch clamp amplifier (BC-700A; Dagan, Minneapolis, MN, USA) was used to obtain membrane potential recordings, where the analog signal was low-pass filtered (cut-off frequency 5 kHz) and digitized at 50–100 kHz (ITC-18, HEKA, Ludwigshafen/Rhein, Germany; RX8, Tucker-Davis Technologies, Alachua, FL, USA). Series resistance was 61.3 ± 3.3 MΩ (mean ± SEM; N = 23, excluding 1 outlier with a series resistance >100 MΩ).

### 2.3. Stimuli

Physiological recordings were done in a double-walled sound-proof booth (IAC, Niederkrüchten, Germany). Tucker-Davis Technologies System II hardware controlled by MATLAB scripts was used to generate/present sound stimuli. Etymotic speakers attached to hollow ear bars were positioned over the ears. The system was acoustically calibrated before each experiment using a probe microphone (Bruel and Kjaer, Nærum, Denmark). Frequency tuning was measured using a threshold-tracking algorithm during contralateral short tone presentation, where spikes were used as triggers. Next, responses to repetitions of short tones were typically collected at the characteristic frequency, presented monaurally contralaterally and if time permitted ipsilaterally (typical parameters: 50–100 ms stimulus duration, 150–200 ms interstimulus interval, 30–50 repetitions at each sound pressure level (SPL) which was varied in steps of 5 or 10 dB). If time allowed, other, primarily contralateral stimuli were presented including click, noise and AM stimuli. In between blocks of stimulus-evoked responses, spontaneous activity was collected. For some cells, responses were also recorded to hyperpolarizing and depolarizing current step injections (duration 100 ms; current amplitude was varied in steps of 100–200 pA).

### 2.4. Analysis

The electrophysiological data were analyzed using custom-made scripts in IgorPro (WaveMetrics, Lake Oswego, OR, USA) and MATLAB (The Mathworks, Natick, MA, USA). Membrane potential values were corrected for the liquid junction potential by subtracting 10 mV from the measured potential (Roberts et al., 2014).

### 2.5. Histology and electron microscopy

At the end of the experiment, the animal was overdosed with pentobarbital and perfused through the heart with saline followed by either paraformaldehyde (PFA) 4 %, or PFA 1 % /glutaraldehyde 1 % or PFA 2 % /glutaraldehyde 1 % all made with 0.1 M phosphate-buffer, pH 7.4. Tissue processing methods for light and electron microscopy have been described previously (Smith et al., 2005) and are briefly summarized here. The brain was removed and stored refrigerated in PFA 1 % /glutaraldehyde 1 % for at least 24 h. 70-μm thick sections of the brainstem were then cut with a vibratome and the biocytin tracer visualized using the DAB-nickel/cobalt intensification method (Adams, 1981). Sections were rinsed in phosphate buffer and these free-floating sections were inspected with a light microscope to determine the location of the labeled cell and, if possible, its axon and dendritic tree.

Some of the sections containing the labeled cell body and relevant portions of its dendritic tree and axon were selected to be processed for electron microscopy (E.M.). Sections not selected for E.M. were mounted on slides, dehydrated, Nissl-stained with cresyl violet and coverslipped for light microscopic evaluation. The outlines of the SOC nuclei in light microscopic images were determined using the Nissl stain. Sections selected for E.M. analysis were fixed in 0.5 % osmium tetroxide for 30 mins, rinsed, and dehydrated through a series of graded alcohols and propylene oxide. Sections were then placed in unaccelerated Epon-Araldite resin and then transferred into a fresh batch of unaccelerated resin overnight. The sections were then embedded and flat mounted in accelerated resin between Aclar sheets at 65 ^◦^C. The region of the plastic-embedded sections containing the labeled portion of the neuron was cut out of the 70-μm section and mounted on the flattened face of a plastic beam capsule. The 70-μm section was re-sectioned into 3-μm sections that were placed on a glass coverslip. The section containing the labeled portion of the cell was selected and removed from the glass coverslip and remounted on a beam capsule. A camera lucida drawing of the section face including the location of the labeled cell part was made and 70- to 80-nm thin sections were then cut and mounted on coated nickel grids. These thin sections were then stained with uranyl acetate and lead citrate and examined using a Philips CM-120 electron microscope.

Measurements of somatic and axon terminal features from electron micrographs were made using ImageJ software (NIH). One feature that distinguishes cell types in other areas of the auditory CNS is the amount of somatic synaptic coverage. To determine percentage of synaptic coverage of a cell body, the length of the surface of the labeled cell was first measured and then the length of apposition of synaptic terminals on the labeled structure was measured in at least 3 sections and averaged. The circumference of each synaptic terminal was also measured.

## 3. Results

### 3.1. General features

We used the gerbil brainstem atlas (Radtke-Schuller et al., 2016) to classify cells as SPN neurons based on their location. More specifically, cells were localized to the SPN based on their distances to surrounding, better defined nuclei. SPN together with LSO, MSO and MNTB all arise at approximately the same rostrocaudal level with SPN situated dorsal and lateral to MNTB, medial to LSO and dorsal and medial to MSO. Progressing rostrally, SPN maintains this position but becomes smaller as the LSO disappears then itself disappears soon after. The validity of our procedure is supported by the observation that the majority of neurons classified as SPN show the physiological responses regarded as characteristic for this nucleus (Magnusson and Gomez-Alvarez, 2019), as documented extensively in the analyses and figures which follow.

**Fig. 1.**
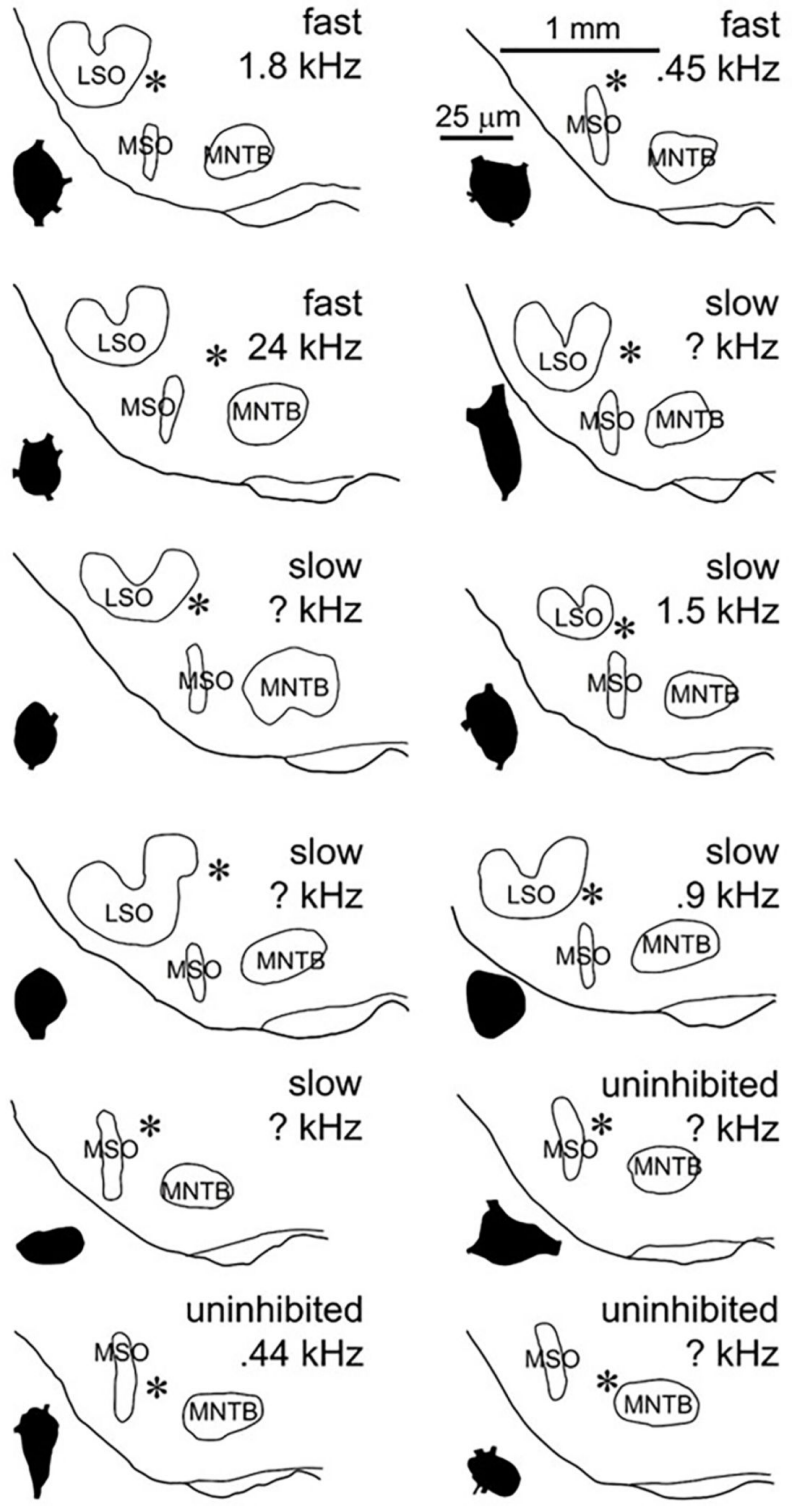
Camera lucida drawings of each cell body (black shape) and its location (asterisks) in the brainstem. Cell type (fast, slow, uninhibited) and CF to contralateral tones (if determined) are indicated. Abbreviations: LSO = Lateral superior olive, MNTB = Medial nucleus of the trapezoid body, MSO = Medial superior olive, kHz = kiloHertz. Scale bars at upper right apply to all figures.

We have obtained whole-cell recordings from and labeled 12 cells in and around this described SPN region (Fig. 1, further discussed in Section 4.2). Although our sample is small, certain response features together with some anatomical criteria have led us to a subclassification of these cells into 3 possible categories. Three of the 12 cells showed no observable inhibitory inputs activated in response to sound and we refer to these as “uninhibited”. These were 3 of the 4 most rostrally situated cell bodies (Fig. 1, bottom: LSO is not visible in these sections). Nine of the 12 cells were strongly inhibited by contralateral sound and displayed the off-responses that are regarded as a hallmark of the SPN. They could be further subdivided into 2 groups based on physiological and anatomical features. Three (out of 9) of these inhibited cells were classified as “fast” SPN cells based on the rapid rate of activation and return to baseline of a contralaterally generated IPSP. In a second group, six (out of 9) cells were classified as “slow” SPN cells based on their much slower rate of IPSP activation and return to baseline.

**Fig. 2.**
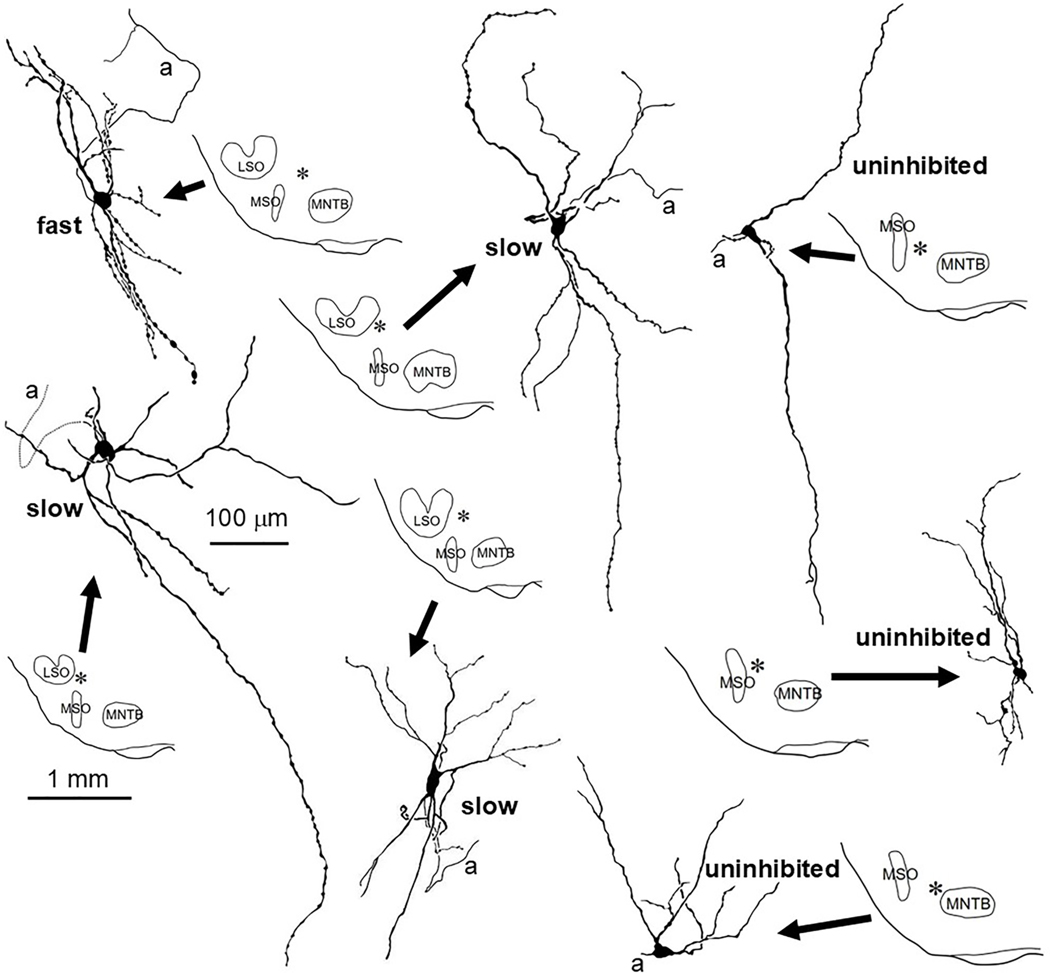
Examples of well labeled fast, slow and uninhibited SPN cells and their brainstem locations (asterisks) relative to LSO, MSO and MNTB. Abbreviations: *a* = axon, LSO = Lateral superior olive, MNTB = Medial nucleus of the trapezoid body, MSO = Medial superior olive. Scale bars apply to all cells/sections.

One fast cell, three of the uninhibited cells and three slow cells were well labeled and are illustrated in Fig. 2. For the others, the cell body was labeled but the dendritic tree was too lightly labeled to draw. Electron microscopy was done on the cell body of 1 fast, 4 slow and 1 uninhibited SPN cell (Suppl. Fig. 1). The fast cell(s) showed extensive synaptic coverage (67.3 %) of the cell body surface, while the 4 slow cell bodies were sparsely covered (8.7 %, 13.3 %, 15.6 %, 35 %). The uninhibited cell body was also sparsely innervated (3.5 %). Examples of synaptic terminals on the 3 cell types are shown in Suppl. Fig. 2. We were only able to follow the axon of one labeled cell far enough to be sure of its innervation target. The axon of this fast cell gave off a local collateral then projected into the ipsilateral lateral lemniscus and faded as it entered the ipsilateral IC.

**Fig. 3.**
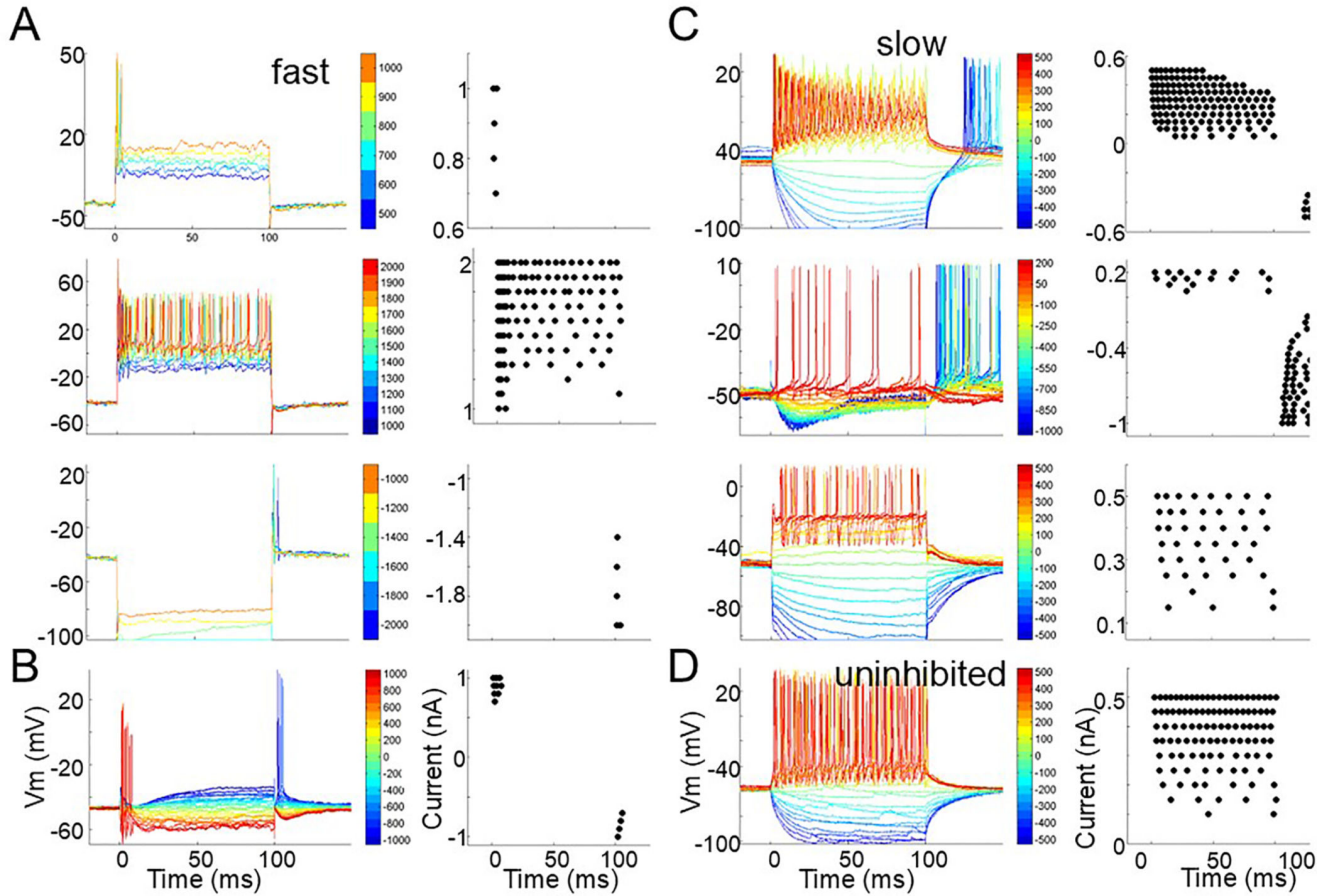
Intracellular responses (left) and spike dot rasters (right) of SPN cells to 100 ms current pulses. A. At low depolarizing current levels (top) this fast cell fired at onset but at higher current levels (middle) the firing became repetitive. Hyperpolarizing pulses (bottom) were followed by a very rapid return to baseline and rapid rebound firing. B. A second fast cell also fired at the onset to low depolarizing currents and rapidly following offset of hyperpolarizing currents. Higher depolarizing current levels were not presented to this cell. C. Three slow cells fire repetitively to depolarizing current pulses even at low depolarizing current levels. Hyperpolarizing pulses were followed by a slower return to baseline and if the cells fired at stimulus offset (top 2 panels) the latency was longer than that seen in fast cells. D. An uninhibited cell also showed repetitive firing at low depolarizing current levels and a slow return to baseline at current offset. Color of trace represents amount of current injected (middle scale). Abbreviations: ms = millisecond, mV = millivolt, pA = picoAmpere, Vm = membrane voltage.

Intracellular recordings during polarizing current step injections (2/3 fast, 3/6 slow, 2/3 uninhibited) also revealed differences between cell types. Fig. 3A and B show the intracellular responses (left) and spike dot raster responses (right) from 2 fast cells. At low depolarizing current levels below 1 nA (Fig. 3A, top trace, B) both cells fired at the onset of a 100 ms pulse but as the current was increased to >1 nA (Fig. 3A, middle trace) the cell responded for the current duration. At the offset of hyperpolarizing current pulses (Fig. 3A, bottom trace, and Fig. 3B) the cell membrane very rapidly repolarized and generated a short latency spike response. These features are in contrast to that seen in cells designated slow (Fig. 3C) or uninhibited (Fig. 3D). At very low depolarizing current levels well below 1 nA both cell types fired repetitively for the duration of the stimulus. In addition, following hyperpolarizing pulses, the membrane returned to rest with a much slower trajectory, and if rebound spikes occurred, they had a longer latency (re. current offset) than the fast cells. As described below, this dichotomy of membrane speed of fast and slow cells was also evident in the membrane response to synaptic inputs driven by sound.

In summary, fast cells show a heavily innervated cell body, only respond at onset to low current levels but can respond repetitively at higher currents and show a rapid return to baseline at hyperpolarization offset and a rapid rebound spike. Slow and uninhibited cells have sparsely innervated cell bodies, fire repetitively even at low depolarizing current levels and can show rebound spikes to hyperpolarizing current offset but at longer latencies due to a slower return of the membrane potential to baseline. Fast cells can project to the inferior colliculus but we do not know where slow and uninhibited cells send their axons.

**Fig. 4.**
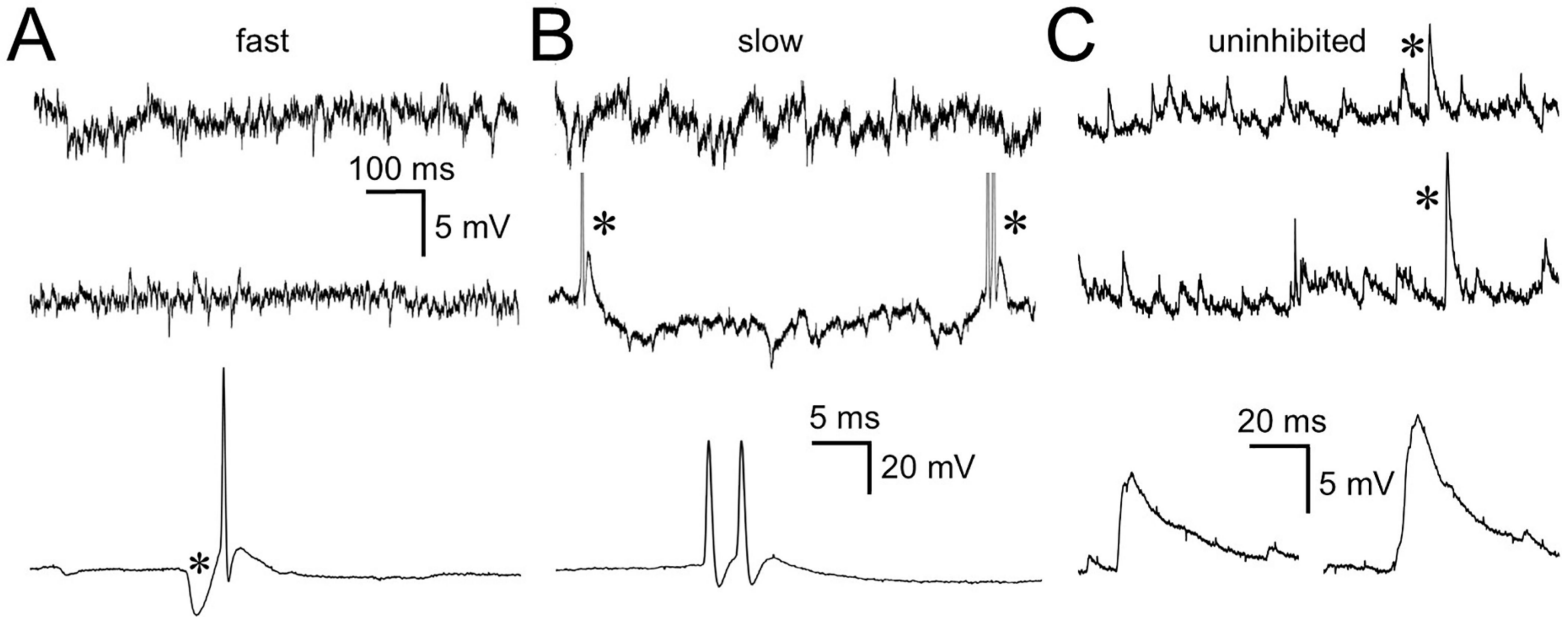
Spontaneous synaptic and spike activity. A. Top 2 traces: fast SPN cell spontaneous synaptic events. Bottom trace: rare spontaneous spike that was always preceded by a large IPSP (asterisk). B. Top trace: slow SPN cell spontaneous synaptic events. Middle trace: examples of spontaneous spikes (asterisks). Lower trace: example of spontaneous 2-spike burst with no preceding IPSP. C. Top 2 traces: uninhibited cell spontaneous synaptic events. Lower trace: time-expanded image showing two examples of large slow EPSPs seen in top 2 traces (asterisks). Scale bar in A applies to top 2 traces in A-C. Bottom scale bar in B applies to bottom traces in A and B. Scale bar in bottom trace of C applies to this trace. Spikes in middle trace of B were clipped to fit.

In the absence of any current or auditory stimulation there appeared to be a low level of both spontaneous excitatory and inhibitory synaptic events for the fast and slow cell types with an occasional spontaneous spike (Fig. 4). For the 3 fast SPN cells, spontaneous spikes were very rare, occurring only once or twice during the entire recording session which could last for 10–20 mins. In these rare cases the spike was always immediately preceded by a large, rapid inhibitory event (Fig. 4A, asterisk) and we presume the spike was generated by a rebound from an inhibitory input from MNTB as has been reported for SPN cells *in vitro* (Felix et al., 2011; Kopp-Scheinpflug et al., 2011). This large rapid inhibitory event was also the hallmark of all fast cell responses to clicks (see Section 3.2, below). For the 6 slow cells spontaneous spiking was more frequent but occurred no more than a few per second, occasionally displaying bursts of 2 or 3 spikes. These spikes were never preceded by a large fast IPSP (Fig. 4B, middle and bottom trace). For the uninhibited cells, although our data are limited, spontaneous EPSPs were present (Fig. 4C) and could sometimes exceed 10 mV (Fig. 4C, lower traces) but even these large EPSPs did not generate spikes. IPSPs were rarely, if ever seen. In summary, all cell types show low levels of spontaneous synaptic inputs and very little spontaneous spiking. When spikes do occur in fast cells they always follow the fast repolarizing phase of a large IPSP. In slow cells the spikes arise from membrane depolarization with no preceding inhibitory event.

### 3.2. Click responses

**Fig. 5.**
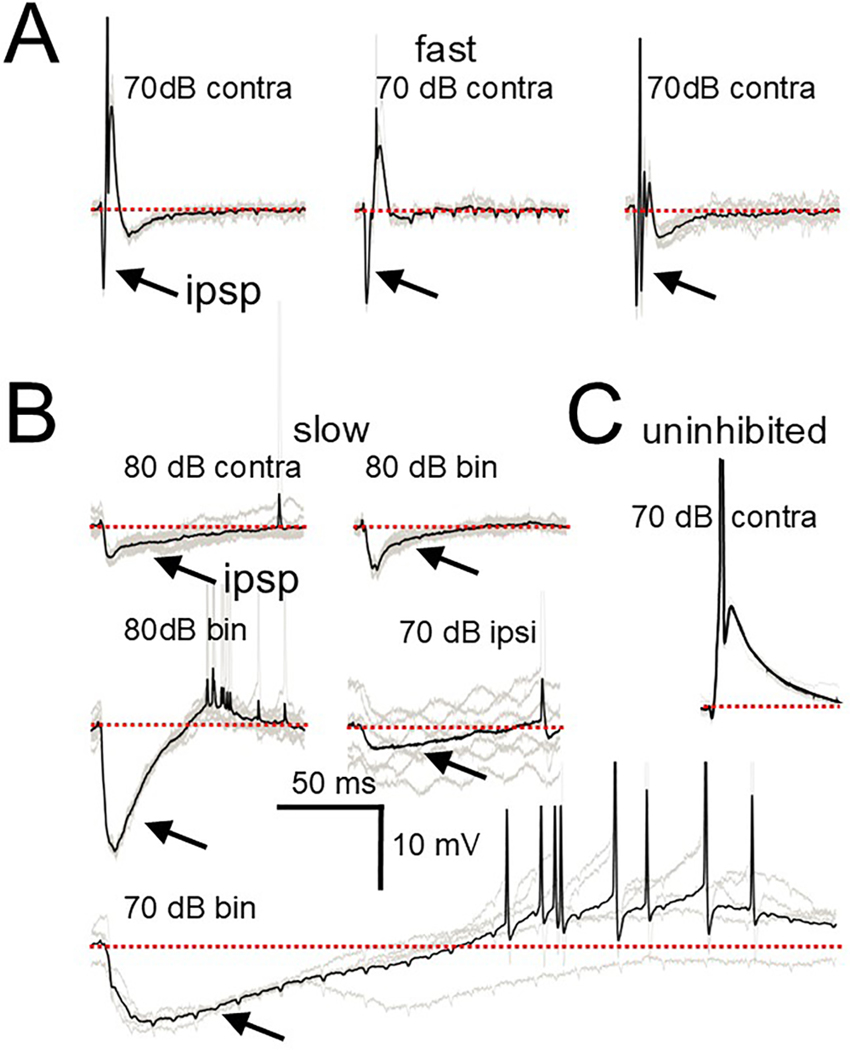
Averaged click response of SPN cells. A. Responses to single clicks in 3 fast cells. A very rapid IPSP (arrow) is followed by a rebound spike. B. Responses to single clicks in 5 slow cells. The IPSP has a slower activation rate than the fast cells and returns to baseline over a much longer time course. C. Response of an uninhibited cell to a single click. In this and the following figures the darker traces are averaged responses of multiple individual trials (lighter traces). Scale bar applies to all traces. Dotted red line is resting potential. Abbreviations: bin = binaural, ipsi = ipsilateral, contra = contralateral.

**Fig. 6.**
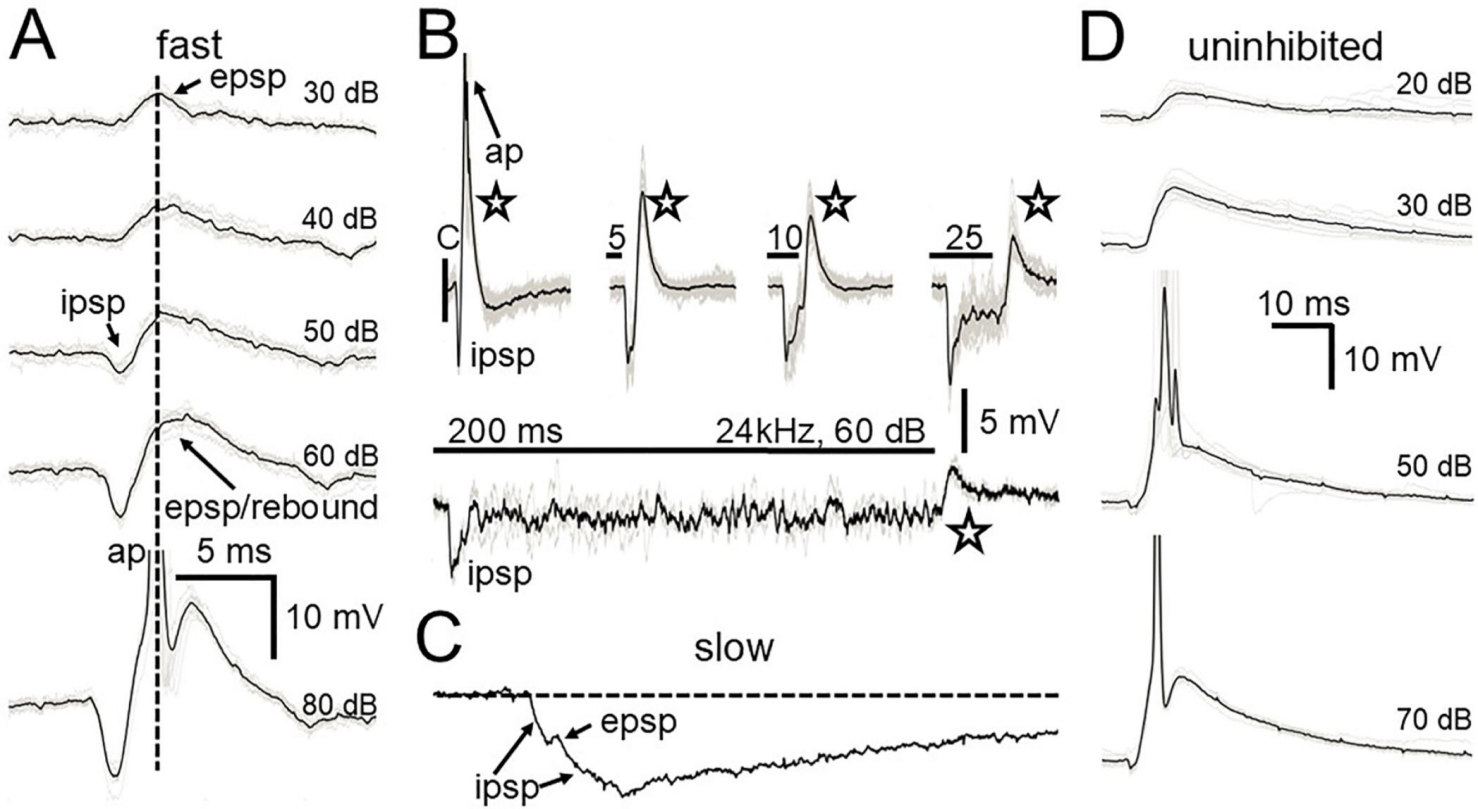
Click responses of fast (A,B), slow (C) and uninhibited (D) SPN cells. A. Averaged responses to a single click at increasing sound levels. For this fast cell the EPSP had a lower threshold than the IPSP (top trace). At increasing sound levels (bottom traces) the IPSP has a shorter latency than the EPSP and generates a rebound depolarization that lines up with the EPSP peak (dotted line) and triggers an action potential (ap). B. Comparison of a fast cell response to click (c) and tone stimuli of different durations. For tones the rebound from inhibition (stars) does not overlap with the short latency EPSP and the response is much less likely to generate a spike. C. Response of a slow cell to a single 60 dB click. The click generated an IPSP and an EPSP, with a shorter latency for the IPSP. Because of the very slow return of the IPSP to rest the EPSP is never able to line up with the IPSP’s rebound to help generate a spike. D. Response of an uninhibited cell to a single click at different sound levels. No IPSPs are seen and the EPSP had a slow rising and falling phase that became suprathreshold at higher sound levels. Scale bars in A applies to traces in A and C. Voltage scale bar in B applies to all traces in B. All spikes are clipped to fit.

To further investigate temporal aspects of the inhibition observed in fast and slow cells, we presented clicks, whose short duration (100 μs) has the benefit of avoiding temporal overlap with the synaptic events that are generated by the stimulus. In response to clicks, all the fast and slow cells tested (3/3 fast, 4/6 slow) showed a strong short-latency inhibitory input driven by the contralateral ear (Fig. 5) presumably from the glycinergic MNTB principal cells that are driven by the contralateral ear and have been shown to project to the SPN (Banks and Smith, 1992; Helfert et al., 1989; Smith et al., 1998; Sommer et al., 1993). These inputs are large and often approached or exceeded hyperpolarization levels of 10 mV from the resting membrane potential, corroborating the results of Rajaram et al. (2020). Rebound spikes were often generated upon repolarization. However, the time course of activation and inactivation of these inhibitory events was much different in the fast and slow categories. Fig. 5A illustrates that for the 3 fast cells, the click-evoked IPSP (arrows) would reach its maximal amplitude in less than a millisecond (0.65, 0.68, 0.69 ms) and then return to baseline within 2.5 ms of this maximum (1.44, 1.94, 2.5 ms). In contrast, for the slow cells the IPSP would peak at least 2.76 ms after onset (Fig. 5B; 2.76, 3.45, 5.67, 5.9, 11.7 ms) and return to baseline over 40 ms later (41, 55, 71, 79, 162 ms). The rebound spike latencies also reflected the fast and slow membrane properties of these categories. Rebound spikes for the 3 fast cells occurred at minimal latencies of 6.7, 7 and 9.8 ms after the click while 3 slow cell rebound spikes occurred with a much longer latency of 46, 66.2 and 260.3 ms. No inhibition was evident in the click response of the uninhibited cell of Fig. 5C (further illustrated in Fig. 6D).

Click stimuli also could generate an EPSP presumably from one or both of the two sources of excitatory inputs from the contralateral CN described in gerbil (Friauf and Ostwald, 1988; Saldaña et al., 2009; Schofield, 1995; Thompson and Thompson, 1991a; Zook and Casseday, 1985). Remarkably, despite the EPSP being a direct CN input and the IPSP an indirect input via the MNTB, the latency of the IPSP was shorter than the EPSP (Fig. 6) for both fast and slow cells, corroborating the fast MNTB mediated inhibition seen in other known targets, the LSO and MSO (Franken et al., 2021; Pecka et al., 2008; Roberts et al., 2013). For the fast cells the rapid rebound depolarization following click-evoked inhibition could coincide with the EPSP (Fig. 6A, dotted line) enhancing the spike probability. For the slow cells the click-evoked EPSP also had a longer latency than the IPSP occurring on the downward deflection of the IPSP (Fig. 6C) but was unable to generate a spike as it did not align with the rebound depolarization following the much slower return to baseline of the IPSP. For the fast cells the temporal overlap of EPSP and rebound from inhibition only occurred for short duration clicks. For longer duration stimuli such as tones (Fig. 6B) this overlap did not occur and the rebound from inhibition was less likely to generate a spike. For the one uninhibited cell tested, a click elicited only an EPSP (Figs. 5C, 6D). At low sound levels subthreshold click-generated EPSPs had a slow time course requiring 50 ms or more to return to baseline. At higher, suprathreshold levels the uninhibited cell would fire one or two spikes at the peak of the EPSP.

**Fig. 7.**
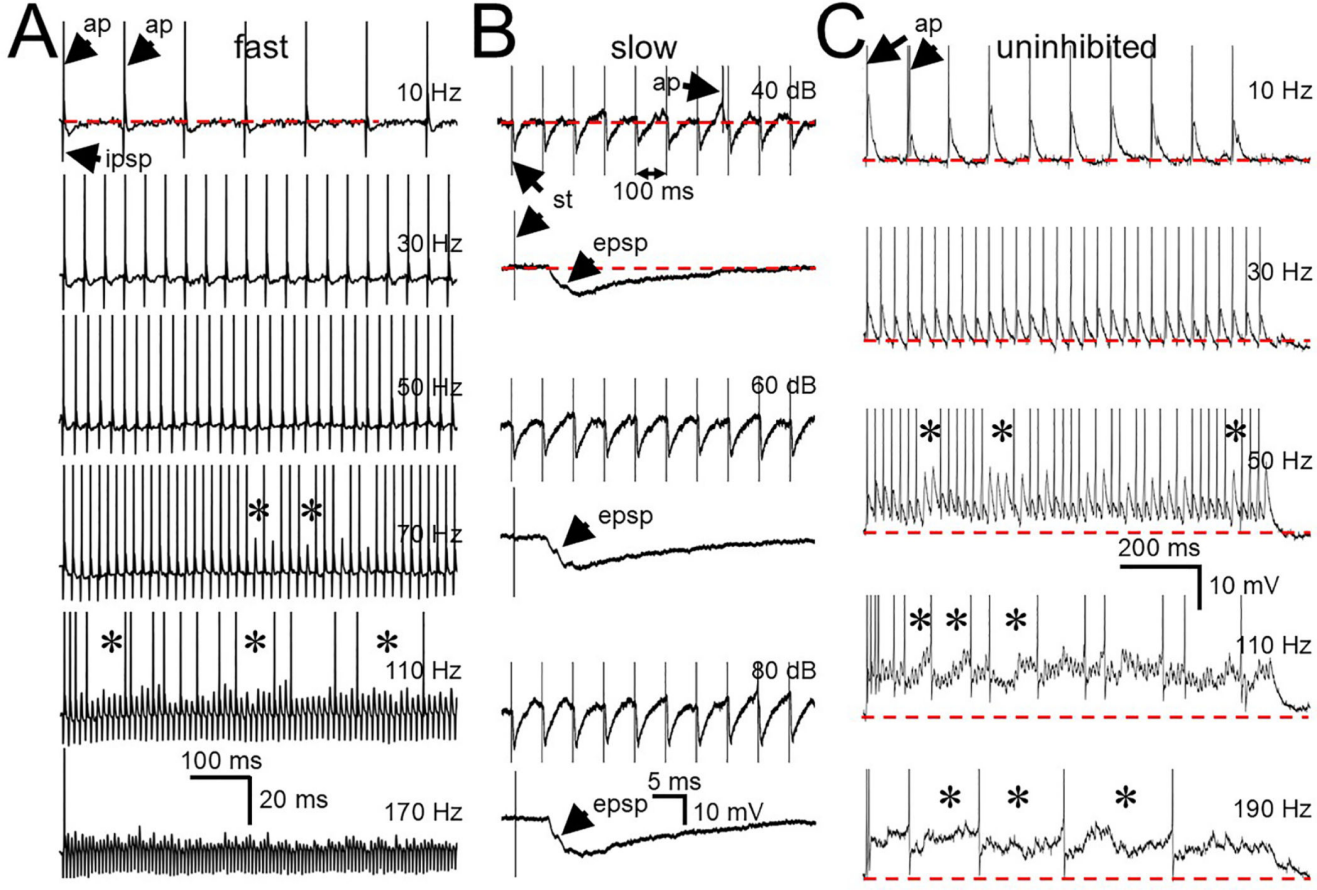
Responses to repetitive click stimuli. A. Single unaveraged responses of a fast SPN cell to click trains at different rates. Rebound spikes (arrows ap), generated by the IPSP could occur at fairly high click rates. Asterisks indicate examples of trials where rebound spiking to every click begins to fail. B. Responses of a slow SPN cell to 10 Hz click trains at 3 different sound levels. 1st, 3rd and 5th traces are responses to 10 clicks; 2nd, 4th and 6th traces are expanded responses to one click. In the expanded response a longer latency EPSP (arrows epsp) can be seen on the downward slope of the IPSP. Even at this low rate the IPSP was only rarely (arrow ap in top trace) able to generate a rebound spike. C. Response of an uninhibited cell at different click rates. Lacking inhibition, EPSPs generated spikes (arrows ap) at fairly high click rates before failing (asterisks). Above 100 Hz, the EPSPs began to sum generating a sustained depolarization and the cell was no longer able to follow the clicks. Dotted red line is resting potential. Spikes are clipped to fit. Abbreviations: st = stimulus.

The membrane speed dramatically influenced the ability of each cell type to follow repetitive click stimuli (Fig. 7). Because of the rapid return of the IPSP to baseline (Fig. 7A) and the subsequent rapid rebound spike (arrows ap), the fast cells were able to follow click trains up to 50 Hz before occasionally missing spike occurrences (asterisks). In contrast, for slow cells (Fig. 7B), even at a click rate of only 10 Hz, the IPSP return to baseline was very slow (expanded traces 2,4,6) and only rarely allowed rebound spiking (top trace, arrows ap) within the 100 ms interval between clicks before inhibition was triggered by the next click in the train. A click-generated EPSP can also be seen on the downward slope of the IPSP (Fig. 7, expanded traces 2,4,6, arrows epsp).

For the uninhibited cell tested (1/3), repetitive clicks only generated EPSPs (Fig. 7C). During click trains the spikes were generated by the EPSPs rather than any rebound from inhibition. In the example shown the uninhibited cell reliably fired to each click up to 30 Hz. At 50 Hz and above the individual EPSPs began to merge and generate a sustained depolarization above rest (dotted line).

In summary, both the fast and uninhibited cells can respond to repetitive click stimuli at fairly high rates due to separate mechanisms (fast repolarization from inhibition or EPSPs in the absence of inhibition). In contrast, the slow cells show a much weaker following ability due to their slow membrane return to baseline causing interference from the successive activation of inhibitory inputs.

### 3.3. Tone responses

**Fig. 8.**
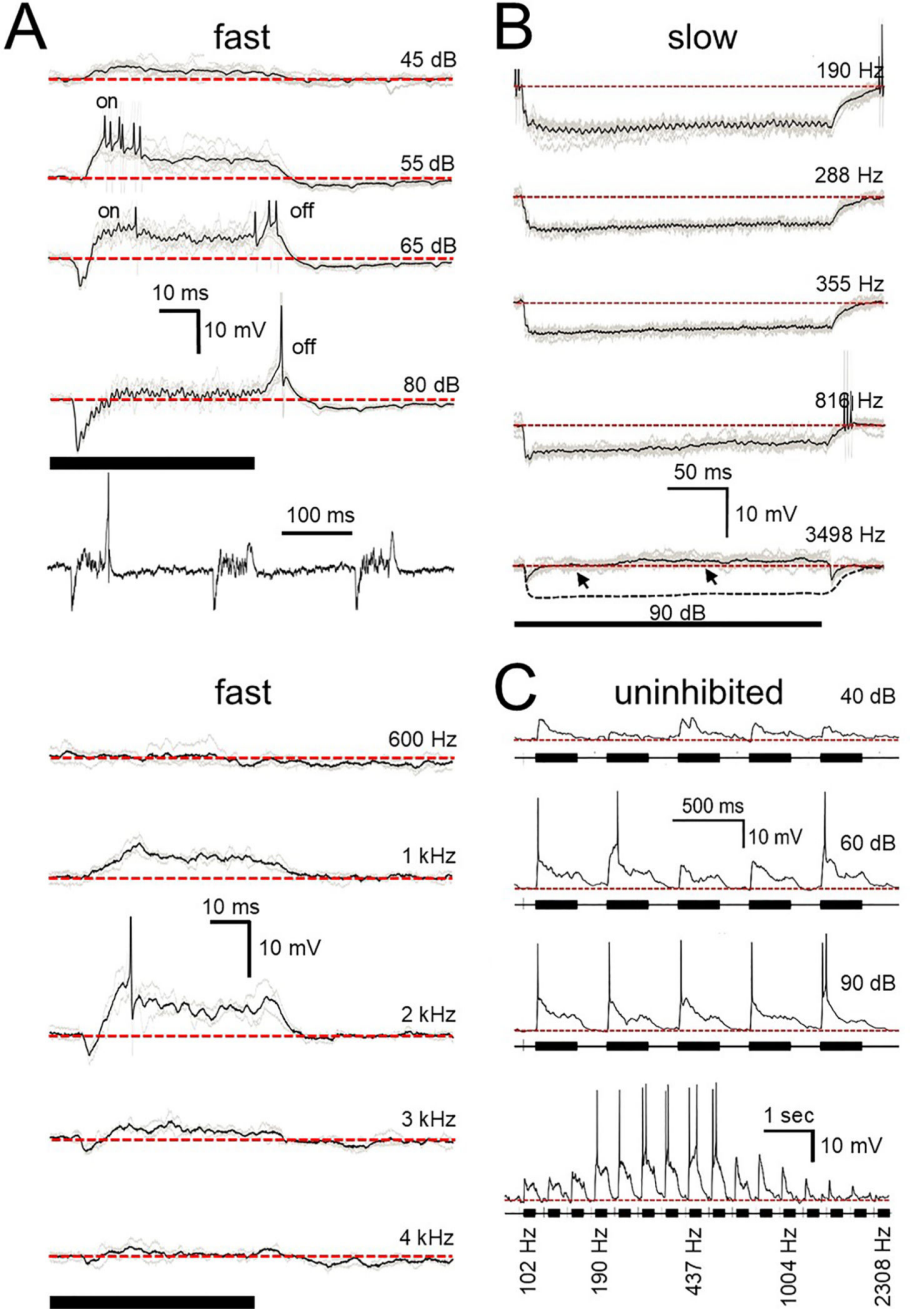
Tone responses of fast (A), slow (B), and uninhibited (C) cells. A. Top fast cell: averaged response to 800 Hz tone at increasing sound levels. At 45 dB only excitation is noted; 55 dB generated onset spikes. At 65 dB inhibition is also activated but the excitation is still capable of generating occasional onset spikes and the excitation combined with the inhibitory offset rebound generated an offset response. At 80 dB the inhibition suppressed the onset response but the offset spiking response persists. Lowest panel illustrates 3 examples of responses to 80 dB tone. Bottom fast cell: averaged responses of another fast cell to different frequencies at the same sound level. B. Averaged slow cell response to a 90 dB tone at various frequencies. At low frequencies a strong, prolonged inhibitory input is activated. At higher frequencies the inhibition is masked by the prolonged excitation (arrows) that is unable to reach spike threshold. Dotted black line in bottom trace is a representation of the synaptic event seen at 355 Hz for comparison. C. Unaveraged response of an uninhibited cell at different sound levels (top 3 traces) and at different frequencies (bottom trace). 10 ms scale bar in A applies to top 4 traces. The 100 ms time scale applies to trace directly below only. Lower 10 ms scale bar applies to all bottom traces. Scale bar in B applies to all traces. Top scale bar in C applies to top 3 traces. Bottom scale bar applies to bottom trace. Dotted red lines indicate resting potential. Spikes in A and B are reduced due to averaging. Thick solid lines in A, B indicate stimulus timing.

As with the click-evoked responses, tone responses of both fast (3/3) and slow (6/6) cells showed considerable inhibition, but they can additionally show excitation that may also play a role in the firing pattern. Fig. 8 illustrates how this balance of excitation and inhibition can vary with sound intensity at a given frequency or at different frequencies at the same sound intensity. The top half of Fig. 8A illustrates the response of a fast cell to CF tones at different intensities. In this cell the excitatory input had a lower threshold than the inhibitory input and generated spikes at onset at 55 dB. Subsequent activation of the persistent inhibitory input at 65 dB suppresses but does not eliminate the onset response and the rebound from inhibition combines with the excitatory input to generate an offset response. At 80 dB the inhibitory input suppresses the onset response and generates a more consistent rebound offset spike response. Thus, depending on the sound intensity this cell could either be onset, offset or on/off. A similar change in the amount of inhibition is seen at different stimulus frequencies at the same sound level, shown in the lower half of Fig. 8A for another fast cell. It is apparent that the amount of excitation varies in a frequency dependent manner. Similar features were seen in the slow cell population where inhibition often dominated but excitation was also present. Fig. 8B shows the response of a slow cell to tones varying in frequency. At low frequencies the response was dominated by inhibition but as stimulus frequency increased a tone-driven excitatory input brought the membrane potential above rest (Fig. 8B, bottom trace, arrows). In our sample the excitation was unable to overcome the inhibitory input and generate a spike. In contrast to the fast and slow cells, uninhibited cells (3/3) show no driven inhibitory input over their entire frequency or intensity range (Fig. 8C). As Suppl. Fig. 3 illustrates, in uninhibited cells the excitatory synaptic events generated by pure tones elicited a slowly activating and inactivating graded EPSP that generated repetitive firing, with no sign of individual phase locked events.

### 3.4. AM responses

**Fig. 9.**
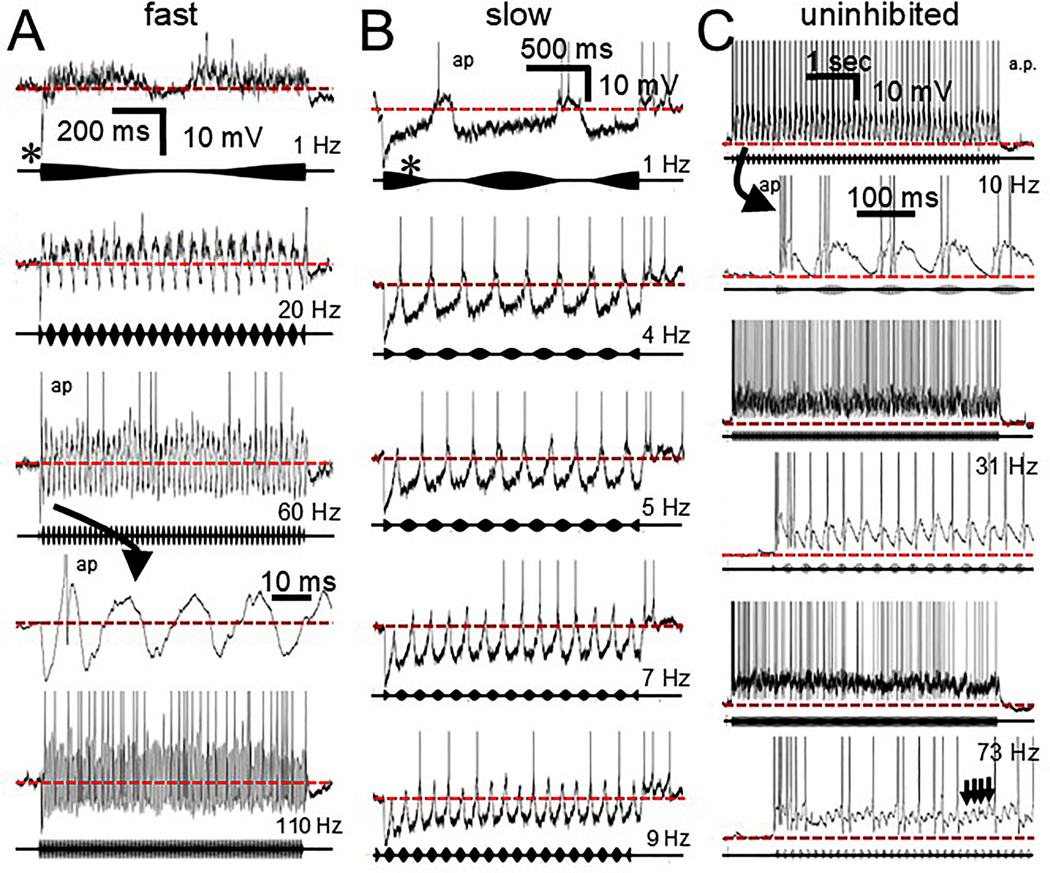
Responses to AM tones at different modulation frequencies. A. Fast cell. Stimulus onset triggers a large IPSP (asterisk). At 1 Hz most of the synaptic input appears to be excitatory. As the modulation frequency increases inhibition is activated and the cell begins to fire rebound spikes (ap). B. Slow cell. Stimulus onset of slowly modulated tones (1 Hz) activates a large onset IPSP (asterisk). Inhibition persists for the duration of the AM stimulus followed by rebound spiking. As modulation frequency increases to 4 Hz the cell follows the envelope with rebound spikes. Above 4 Hz the return to baseline of the IPSP is in some cases too slow to generate a spike before the next cycle is initiated. C. Uninhibited cell. At 10 Hz, each modulation cycle elicits a slow sustained depolarization (arrow and inset) and multiple spikes. As modulation frequency increases to 31 Hz the cell begins to display a sustained depolarization (expanded trace) but is still able to follow the modulation with a spike. At 73 Hz the cell still shows phase locked EPSPs (arrows) but many of the spikes are generated instead by the sustained depolarization (expanded trace). Scale bar in A applies to all traces except the 4th trace which is a temporally expanded version of the beginning of the 3rd trace (curved arrow). Scale bar in B applies to all traces. In C the 2nd 4th and 6th traces are temporally expanded versions of the onset of responses in the 1st 3rd and 5th traces. Dotted red line = resting potential. Spikes are clipped to fit. Carrier frequency was at CF.

When tested in 1/3 fast, 4/6 slow and 2/3 uninhibited cells all three response types fired in a phase-locked fashion to the envelope of AM tones. As with the responses described above for clicks and tones, the presence or absence of inhibition and the speed of the membrane response to the synaptic input played important roles (Fig. 9). For the fast cells (Fig. 9A) the onset of a low-frequency modulator generated a large inhibition (asterisk) but the envelope itself was unable to activate the inhibitory input. As the modulation frequency increased about 5–10 Hz a strong rapid IPSP was generated for each cycle causing rebound firing which increased in reliability as the AM frequency approached 100 Hz. This was possibly because of the ability of the cell membrane to rapidly return and overshoot the resting potential generating a spike. Above 110 Hz the spike reliability began to decrease. For the slow cells (Fig. 9B) a large inhibition was generated by the stimulus onset (asterisk) but, at low modulation frequencies, inhibition and a slow return of the membrane potential was also seen which overshot the resting potential and generated offset spiking. At a modulation frequency of 5 Hz and higher the rate of return of the membrane potential became too slow and the overshoot was interrupted by the hyperpolarization generated by the following cycle of the signal. As a result, spike reliability rapidly declined with increasing modulation frequency. For the uninhibited cells (Fig. 9C) the response to AM signals was totally dependent on the EPSPs generated. At 10 Hz the cell elicited a large slow EPSP and multiple spikes (curved arrow) and the envelope period was long enough that the membrane potential could return to rest after every cycle. With increasing envelope frequency the membrane potential did not return to rest after each cycle (red dotted line) but the EPSP peaks were still able to generate spikes that were locked to each cycle. Above 50 Hz, phase locked EPSPs could still be seen (lower trace, arrows) but the cell began to fire to the sustained depolarization and phase locking to the envelope diminished.

### 3.5. Noise

**Fig. 10.**
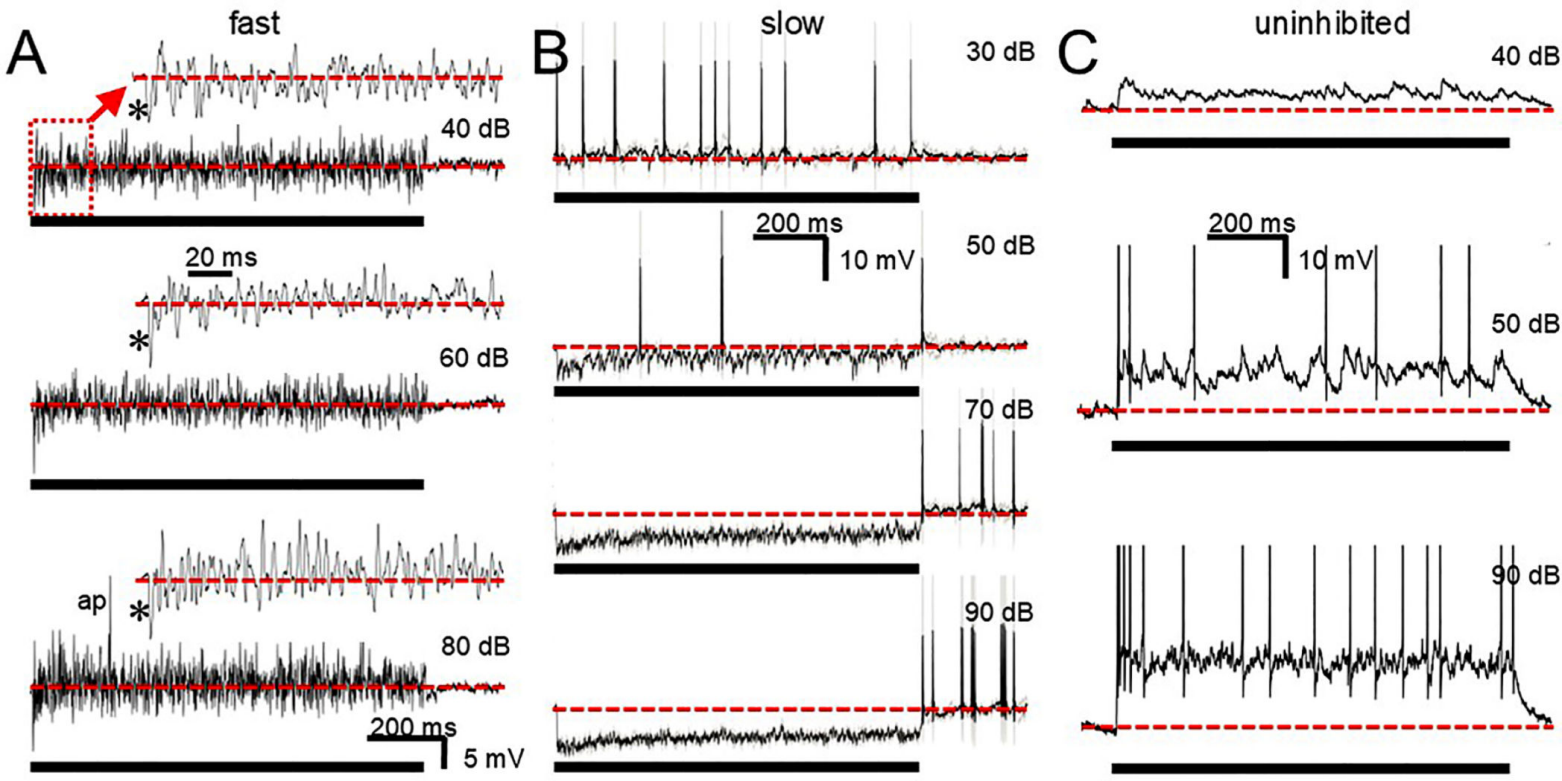
Noise responses at increasing sound levels. A. Individual traces from a fast cell that appears to generate a blend of EPSPs and IPSPs for the stimulus duration with an occasional action potential (ap). Insets (arrow) are temporally expanded versions of the beginning of the trace below: time scale in second inset applies to all insets. A large fast IPSP is consistently generated at onset (asterisks). B. Averaged slow cell response shows excitation and spiking at low sound levels but with increasing stimulus intensity begins to respond at the offset of inhibition. C. Individual traces from an uninhibited cell that responds with a prolonged, sustained depolarization that generates several spikes. Scale bars in A and B apply to all traces in the respective columns. Scale bar in C applies to all traces. Heavy black bar in A,B,C indicates noise duration. Dotted red line indicates resting potential. Spikes in B are reduced due to averaging. Spikes in C are clipped to fit.

We also presented noise to a number of cells (3/3 fast, 4/6 slow, 2/3 uninhibited). For both the fast and slow cells, noise could generate both excitation and inhibition. Fig. 10A illustrates a fast cell. At the onset of the noise the typical large fast IPSP is apparent (asterisks) which is then followed by what appears to be a combination of IPSPs and EPSPs that only rarely generate a spike (ap). The slow cell shown in Fig. 10B displayed noise driven excitation in the apparent absence of inhibition at low SPLs (top trace), that was able to drive the cell. As SPL increased (lower 3 traces) persistent inhibition suppressed the excitation and was followed by offset firing. Thus, depending on stimulus strength, the cell response could be sustained or offset. For another slow cell (not shown) the threshold for inhibition was lower and the cell always responded with offset spikes. For the uninhibited cells (Fig. 10C) only excitatory events are seen at any sound level and the noise generates a persistent depolarization and spiking.

### 3.6. Gap detection

Brief soundless gaps in auditory stimuli are thought to be a major cue for vocal communication (Walton, 2010). Others (Kadner and Berrebi, 2008; Kopp-Scheinpflug et al., 2011) have indicated that certain SPN cells are capable of detecting such gaps in auditory stimuli down to a few milliseconds or less thanks to the ability to rapidly rebound from inhibition and fire. Although our data are limited (N = 2) it would appear that the slow cells are incapable of detecting such brief gaps. Suppl. Fig. 4 shows the response of one slow cell to 200 ms tone stimuli with increasing inter-tone gaps. At gaps of 40 milliseconds or greater (bottom 2 traces) the rebound from prolonged inhibition can generate a spike. Below this gap duration (top 2 traces) the slow rebound from inhibition is unable to generate a spike before the onset of the next inhibitory event. Unfortunately, we did not present similar stimuli to the fast cells so we do not know whether they would be capable of rebound firing at smaller gaps. However, this seems likely given their more rapid return to baseline and their ability to follow/rebound from clicks with 10 ms intervals.

### 3.7. Duration coding

**Fig. 11.**
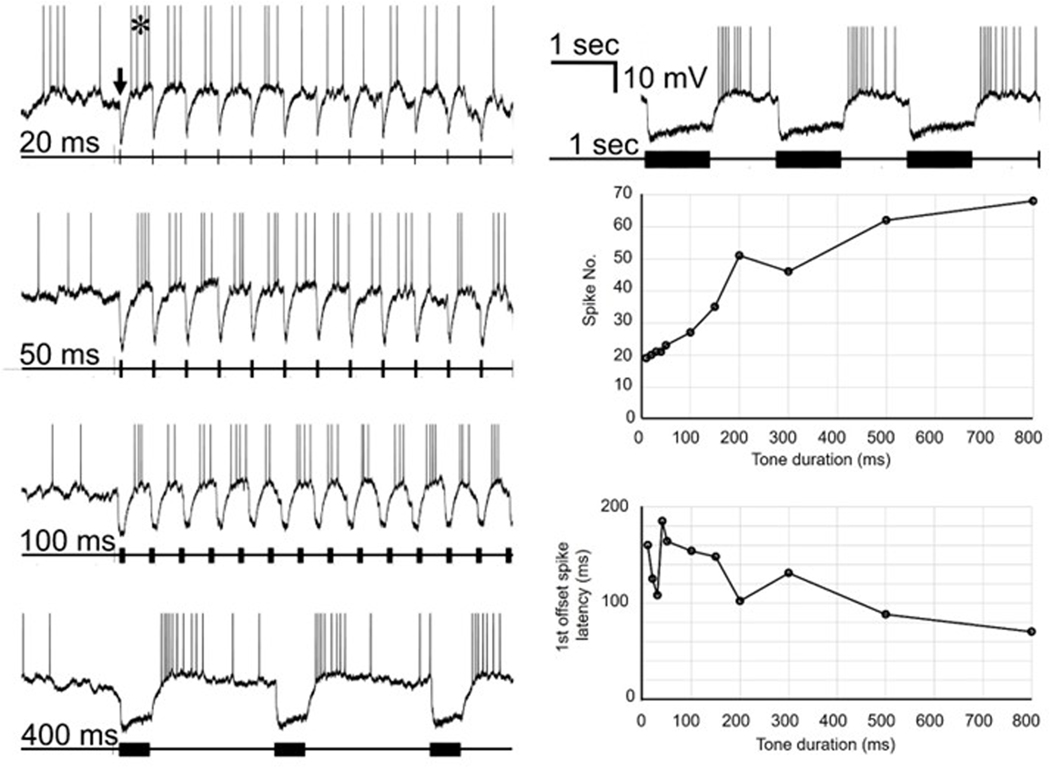
Effect of tone duration on response of slow cell. Left column and top trace in right column: rebound spiking in response to CF tones of increasing duration. Scale bar in top right trace applies to all traces. Arrow and asterisk in top trace (left) indicate inhibitory response and rebound spiking. Middle panel right column: number of rebound spikes in response to 10 tones of different durations. Bottom panel right column: average latency of the first spike to 10 tones of different durations.

In rat (Kadner et al., 2006; Kulesza et al., 2007) some SPN neurons are capable of coding stimulus duration based on the latency and number of rebound spikes generated after stimulus offset. As the stimulus duration increases, the number of spikes increases and the latency of the first spike decreases. We presented tones of different duration to one of the slow cells (Fig. 11). Tones of all durations generated a persistent hyperpolarization followed by rebound firing (Fig. 11, left column, top trace in right column). Although the latency to the first rebound spike was long (right column, lower plot) there was a tendency for the latency to decrease as the tone duration increased. This was accompanied by an increase in spike number (right column, middle plot). Once again, unfortunately we did not replicate this experiment on any of the fast cells.

### 3.8. Ipsilateral inputs and binaural interaction

**Fig. 12.**
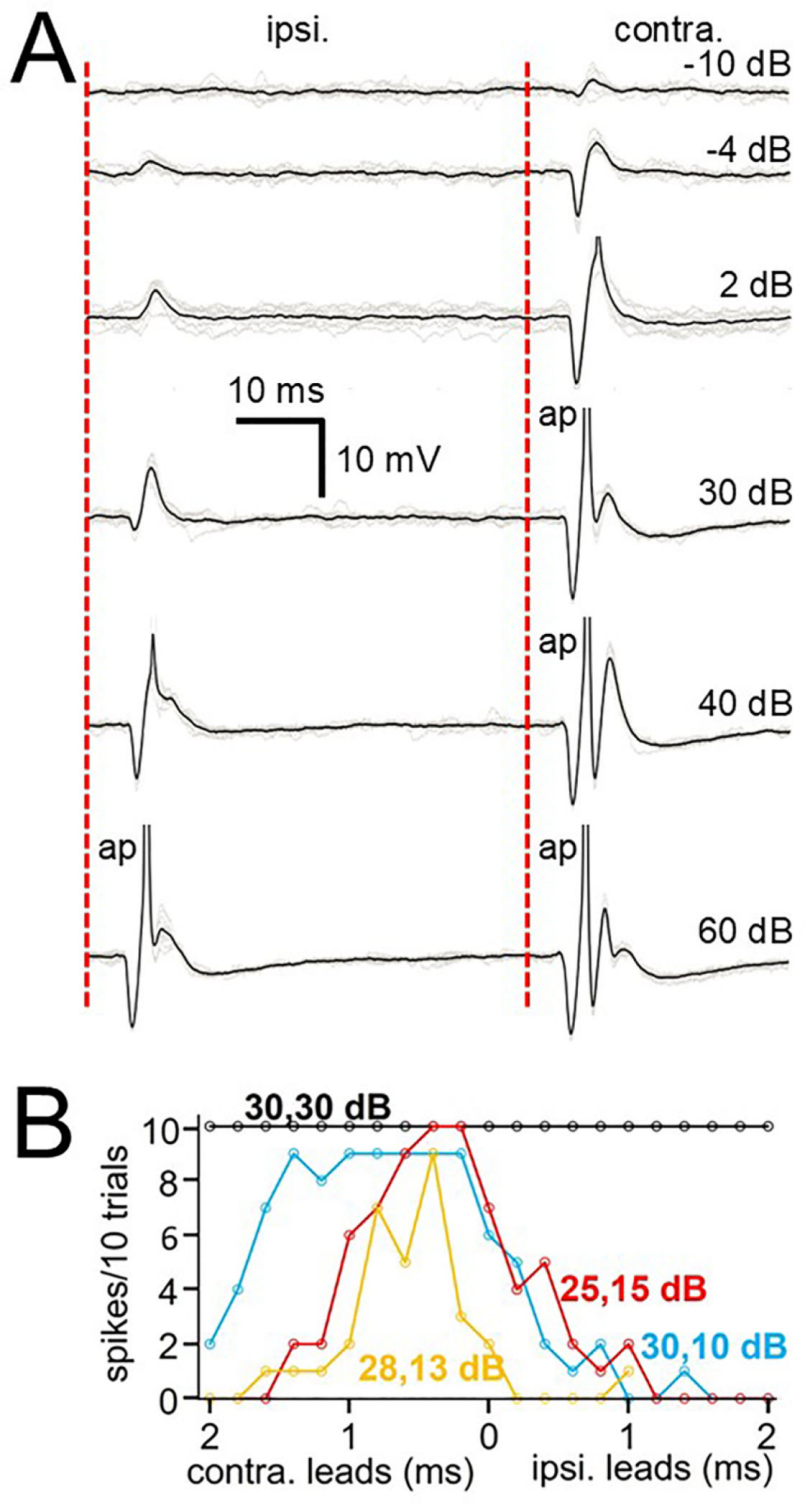
Fast-cell responses to ipsi- and contralateral clicks and binaural click delays. A. Response of the cell to ipsilateral (left) and contralateral (right) monaural clicks at increasing sound levels (contralateral click threshold = - 10 dB; ipsilateral click threshold = - 4 dB). Dotted vertical line is time of click occurrence. Acoustic crosstalk possibly contaminates the results at 40 and 60 dB. B. Plot of the cell’s spike output at various sound levels as the interaural time delay (ITD) was varied from 2 ms leading in the contralateral ear to 2 ms leading in the ipsilateral ear. Scale bar in A applies to all traces. Colored ipsi and contra sound levels are indicated for the plot of the same color.

**Fig. 13.**
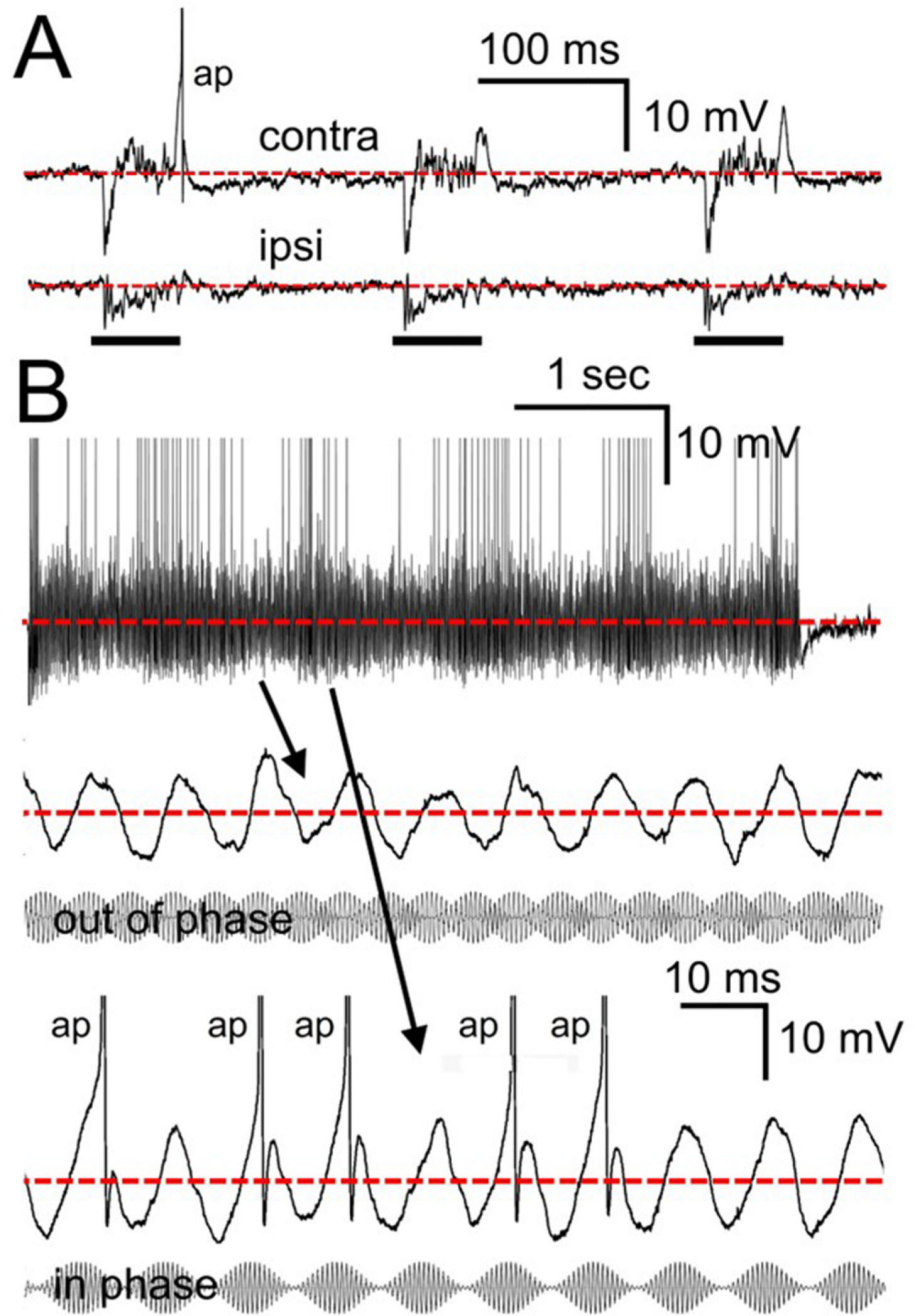
Fast-cell responses to binaural AM stimuli. A. Responses to 3 presentations of monaural contralateral and ipsilateral 50-ms tones at CF (=1.8 kHz) (contralateral tone = 80 dB, threshold = 40 dB; ipsilateral tone = 90 dB, threshold = 60 dB). B. Response to a 5-s binaural AM beat stimulus at modulation frequencies of 101/102 Hz, carrier = 1.8 kHz, 55 dB. Expanded traces below illustrate synaptic events and spikes (ap) during in and out of phase portions of the stimulus. When the stimulus envelopes are in phase at the two ears the rebound from inhibition is also in phase resulting in a larger, more well-defined peak. The red dotted line represents the resting potential. Upper scale bar in B applies to top trace, lower scale bar to expanded middle and lower traces. Spikes are clipped to fit.

During the usually brief intracellular recording sessions we focused first on the cell responses to contralateral inputs and then, when possible, on ipsilateral and/or binaural inputs. For fast cells we were able to present ipsilateral tones and clicks to 3/3 cells, noise to 1/3, AM tones to 1/3 and binaural AM and tone beats to 1/3 cells. For slow cells we presented ipsilateral tones to 5/6 cells, clicks to 4/6, noise to 3/6, AM to 2/6 and binaural stimuli to 2/6 cells. For the uninhibited cells we only presented ipsilateral tones, clicks and noise to 1/3 cells. As a general rule, for each of the cell types, the ipsilateral inputs tended to resemble the contralateral input in that the fast and the slow cells usually showed both excitatory and inhibitory ipsilateral inputs although they were typically not as large. The uninhibited cells showed only ipsilaterally generated excitation similar to but much reduced in amplitude when compared to contralateral inputs. Fig. 12 illustrates click responses for one of the fast cells to both ipsilateral and contralateral clicks. As the sound level increased, both ipsilateral and contralateral responses showed a large, short latency IPSP followed by a rebound spike (Fig. 12A). We then presented binaural clicks at different interaural time delays (ITDs) and sound levels (Fig. 12B). As seen in the plots, for most sound levels the cell showed ITD-sensitivity, preferring ITDs where the sound leads in the contralateral ear. Another fast cell responded to ipsilateral tones at CF with onset and sustained inhibition and what appeared to be excitatory events riding in the inhibition (Fig. 13A) similar to the contralateral response to the same tone but much reduced in amplitude. When presented with AM tones (101 and 102 Hz) with the carrier frequency at CF the cell showed a response weakly locked to the binaurally beating envelope (Fig. 13B, top trace) responding preferentially when the envelopes were in phase (Fig. 13B, bottom traces).

**Fig. 14.**
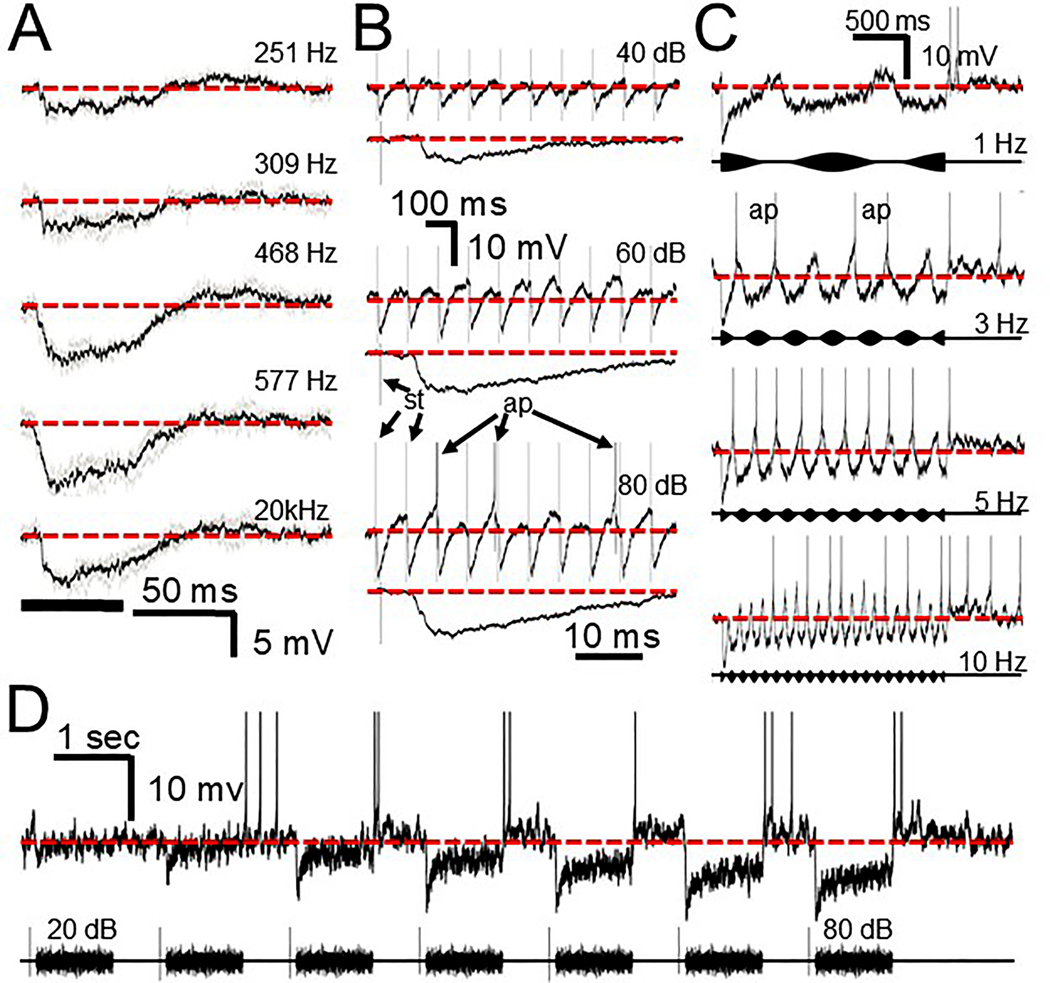
Slow-cell responses to a range of ipsilateral sounds. A. Averaged responses to a single 50-ms, 90-dB ipsilateral tone at different frequencies. B. Individual responses to 10-Hz ipsilateral click trains at different sound levels. Rebound spikes (ap) occur at 80 dB. Responses to a single click are expanded in traces 2, 4 and 6. Vertical grey lines (arrows st) show the stimulus clicktrain superimposed on the responses. C. Individual responses to 1 to 10 Hz AM using a 700-Hz carrier. D. Individual response to a 1-s noise burst at increasing sound levels. The vertical gray lines preceding each noise burst are timing signals not transmitted to the speaker. Scale bars in A-D apply to all traces in the respective panels. Dotted red line = resting potential. Spikes are clipped to fit. Levels of the ipsilateral stimuli for each of the presented stimuli did not exceed values that might generate crosstalk.

We presented ipsilateral tones, noise, clicks and AM stimuli to several slow cells. Inhibition dominated the response to ipsilateral tones, presented over a range of frequencies (Fig. 14A) as well as to click trains (Fig. 14B), AM tones (Fig. 14C), and noise (Fig. 14D), all presented over a range of sound levels. Except for the pure tones, all these stimuli generated rebound spikes.

For the uninhibited cells we have one example of a response to ipsilateral clicks, noise and tones. All the synaptic events are excitatory just like the contralateral input (Suppl. Fig. 5) but again the amplitudes were comparatively reduced.

## 4. Discussion

We have recorded intracellularly from and labeled cells in the gerbil SPN region *in vivo*. Our main result is that we could classify cells into 3 categories: fast, slow and uninhibited, based on anatomical and physiological data. Despite the limitations inherent to our recording and labeling technique (small sample size; limited time of physiological exploration), the proposed classification revealed key differences along multiple dimensions including ultrastructure, current injection, and response to sound, and brings a synthesis to disparate findings from previous reports addressing the anatomy, in-vitro physiology, and in-vivo extracellular physiology of this nucleus. We first summarize our findings, to then provide a more detailed discussion relating our findings to previous reports.

### 4.1. Cell classification

The major distinguishing feature of these cell classes is the presence or absence of inhibition driven by contralateral sound and, if present, its influence on the cell’s response. Both fast and slow cell categories receive large, short latency sound-driven inhibition that often generates a rebound spike, however the time course of the IPSP and the latency of the rebound spike is more rapid in fast cells. Uninhibited cells display no observable inhibitory inputs activated by the auditory stimuli we presented. All 3 cell types also receive sound-driven contralateral excitatory inputs. For the uninhibited cells this is the sole contralateral input and determines the spike output of the cell. For the fast and slow cells this excitation appears to play a much more minor role in the driven auditory responses. Several other features distinguish the cell classes. In response to passive current injection the slow and uninhibited cells fire action potentials repetitively for the duration of the stimulus even at low current levels. By contrast, fast cells fire only at onset at low current levels but as current levels increase begin repetitive firing. Note that none of these types correspond to the bursting cells that have been identified in the SPN of mice P12 or younger (Felix et al., 2013). In response to hyperpolarizing currents both fast and slow cells can generate rebound spikes but, as with spikes generated by rebound from sound-evoked inhibition, the latencies are shorter for the fast than for the slow cells. Anatomically the dendritic trees of all cells are multipolar. At the E.M. level the cell bodies show differences in synaptic coverage with dense coverage of fast and sparse coverage of slow and uninhibited cell bodies. The axon of a fast cell projected to the ipsilateral IC and had a local SPN collateral but we do not know which cell type is influenced by these collaterals, where the slow and uninhibited cell axons project, and whether these have local collaterals.

### 4.2. Anatomy

In rats and mice injections of retrograde tracers into the IC labels a large percentage of SPN cells ipsilaterally (Saldanã and Berrebi, 2000; Willard and Ryugo, 1983) leading some to the conclusion that in these species possibly all SPN cells project to ipsilateral IC (Saldaña and Berrebi, 2000). In contrast, guinea pig SPN houses neurons that can project either to IC or to CN with cells projecting to IC tending to have large round cell bodies and multipolar dendritic trees and CN projecting cells tending to have smaller, more elongate cell bodies (Schofield, 1991). We are hesitant to characterize cell body features in cells that have been patch clamped as it is our experience that cell shape can be altered by prolonged intracellular recordings with patch electrodes. Regarding dendritic tree configuration, as Figs. 1 and 2 illustrate, all three cell types have multipolar dendritic trees which can extend for hundreds of microns away from the cell body without extensive branching. Because our N is small, we are hesitant to make any quantitative distinctions between groups.

In the gerbil a large number of cells have been reported to project to the ipsilateral IC (Nordeen et al., 1983) however no data is available to indicate whether, as in guinea pigs (Schofield and Cant, 1991), a second SPN population(s) exists that instead projects to CN. A feature of mice SPN cells appears to be the heavy innervation of the cell body primarily by glycinergic terminals and a few glutamatergic terminals (Rajaram et al., 2019, their Fig. 1). These combined reports would thereby imply that, in the mouse, IC-projecting SPN cell bodies are covered with synaptic terminals. Our data in gerbil also shows that the SPN cell whose axon projected to the IC had a cell body that was heavily innervated. This cell was one of those designated fast. The slow and uninhibited cell bodies in contrast were sparsely innervated. We might then speculate that the gerbil is more like the guinea pig, with one population that projects to the IC with a heavily innervated cell body and one or more populations that project to the CN with sparsely innervated cell bodies. A further bit of evidence that it is the fast cells that project to the IC is from our axonal recordings in the lateral lemniscus, the pathway of ascending inputs headed to IC, which in chinchilla is readily approachable via the middle ear (Bremen and Joris, 2013). Here all recordings with SPN physiology resembled the fast cell features we have reported here (Joris and Smith, unpublished).

As described above, one of the axons of a fast cell gave off a local collateral that branched within the SPN as the main axon headed to the IC. GABAergic terminals have been reported in the SPN (Kulesza and Berrebi, 2000) and GABA antagonists have been shown to have an effect on SPN cell responses (Kulesza et al., 2007) leading the authors to speculate that this GABAergic inhibition arose from collaterals of SPN cells. Our data shows that the fast SPN cells are likely the source of at least part of this inhibition. Whether this input influences one, two or all types of SPN neurons remains to be determined. A lack of observable driven inhibition in the uninhibited class would suggest that this class is not influenced.

The borders of SPN are ill-defined and vary across studies, even within the same species. A strong argument in support of our assignment of labeled cells to the SPN is that 9 of the 12 cells showed strong, short-latency inhibition to contralateral sound: a feature that is seen as a hallmark of this nucleus (Magnusson and Gomez-Alvarez, 2019). This feature is not present in the 3 uninhibited cells. These cells are physiologically and morphologically too different from MSO and MNTB neurons to consider them (displaced) cells from those nuclei. One may speculate that, in view of their rostral position (Fig. 1), possibly these cells indicate a separate grouping or (sub)nucleus.

### 4.3. Intrinsic membrane features

We presented current pulses to a limited number of cells. During *in vivo* recordings, careful adjustment of the electrode bridge balance is not always feasible or reliable so some of the recordings show rapid transients at the onset and offset of the current pulse that are the unbalanced response of the electrode and not the response of the cell membrane (Fig. 3). Regardless, the data still indicate that the fast cell membrane tends to polarize rapidly at the abrupt onset and offset of current pulses and to fire only at onset to low depolarizing currents but repetitively at higher current levels (Fig. 3A). A similar “fast” membrane response was reported for post-hearing onset mouse SPN cells in brain slices with most displaying onset spiking at low current level that often became repetitive at higher current levels (Felix et al., 2013): again this suggests that the fast cells in gerbil SPN correspond to all the SPN cells in mouse. The slower membrane features and repetitive firing even at low current levels that we see in the slow and uninhibited gerbil categories (Fig. 3C, D) were not reported in mouse but as described above these cells may not be represented in the mouse SPN.

Cells with “fast” membranes are frequently encountered in auditory brainstem circuits and are associated with the system’s “need” to be able to code and extract both monaural (bushy and octopus cells) and binaural (MSO and LSO principal cells) temporal cues with great precision. Here, the membrane of fast SPN cells may be dedicated to the generation of a fast rebound from inhibition. This permits these cells to rebound coincidentally with a click generated EPSP or to give a well-timed estimate of the offset of, or gap in, an auditory event.

### 4.4. Inhibitory synaptic inputs

In most species it is thought that 2 excitatory and one inhibitory contralateral input driven from the contralateral ear act as the primary regulators of SPN cell activity. The inhibition arises from the glycinergic MNTB cells on the same (homolateral) side as the SPN that are driven by globular bushy cells in the contralateral CN. In mouse around 4 MNTB cells are reported to converge on a single SPN cell generating a very large IPSP thanks to the very negative chloride reversal potential (Kopp-Scheinpflug et al., 2011; Löhrke et al., 2005; Rajaram et al., 2020). Both our fast and slow cells in gerbil also could show a very large contralaterally elicited IPSP (Figs. 5–9), so we would assume that both cell types have this very negative chloride reversal potential.

Oddly, both the fast and slow cells could also receive inhibitory inputs driven by the ipsilateral ear (Figs. 12–14). These were typically much weaker than those seen upon contralateral stimulation. Although excitatory inputs driven by the ipsilateral ear have been reported (see next section), no reports of an inhibitory input are described in the literature, so the origin of this inhibition is unclear.

### 4.5. Excitatory synaptic inputs

There is some disagreement on the source(s) of the major contralateral excitatory inputs to the SPN (Magnusson and Gomez-Alvarez, 2019). In most species it is fairly well established that octopus cells in the contralateral CN provide what is considered a strong excitatory input to the SPN by way of their axons which run in the intermediate acoustic stria (IAS) (Smith et al., 2005). In slices, when glycinergic and GABAergic inputs are blocked and the IAS is shocked, large fast glutamatergic EPSPs are generated presumably from these octopus cell inputs (Felix and Magnusson, 2016). *In vivo*, octopus cells typically respond to tones with an abrupt, well timed onset spike and little or no sustained spiking and to clicks with a similar single well-timed spike (Godfrey et al., 1975; Lu et al., 2022, 2018). To click trains, octopus cells show entrained, remarkably well-timed spiking up to several hundred Hz in gerbil (Lu et al., 2022, 2018) and even higher in cat (Godfrey et al., 1975; Oertel et al., 2000). So, one would imagine that excitatory synaptic events generated in response to tones in a cell receiving an octopus input would occur at stimulus onset with little or no sustained activity. We did see early EPSPs generated by contralateral clicks and tones in the slow cells; however, the EPSP onset was typically preceded by the large IPSP (Figs. 6C, 7B) arising from MNTB inputs. This is somewhat surprising because the IPSP is generated through a circuit (bushy cell to MNTB cell to SPN cell) that includes an extra synapse. The resulting placement of the EPSP on the downslope of the IPSP rendered the EPSP subthreshold which is probably not remarkable because it has been established in mice (Rajaram et al., 2019) that the current amplitude and conductance generated by the IPSP is greater than the EPSP current amplitude and conductance generated by the octopus cell input. It is also of note that octopus cell spike thresholds are considerably higher than bushy cell thresholds (Lu et al., 2022; Rhode and Smith, 1986) so it is likely that the bushy-cell-driven inhibitory MNTB input would be activated at lower SPL levels than excitation driven by octopus cells. A similar early EPSP can be seen in the fast cells but again its latency is longer than the large MNTB-generated IPSP (Fig. 6A). For click stimuli this seems to be advantageous for fast cells in that the EPSP lines up with the rapid IPSP rebound making it more likely for a spike to be generated. However, for longer duration auditory events like tones the EPSP effect is nullified by the sustained inhibition (Fig. 6B).

The finding of shorter-latency inhibition (re. excitation) in SPN neurons adds to a growing list of brainstem neurons where such temporal relationship has been documented including commissural cells in posteroventral CN (Joris and Smith, 1998), principal cells in MSO and LSO (Franken et al., 2021; Roberts et al., 2013), and ventral nucleus of the lateral lemniscus (Nayagam et al., 2005).

The second direct excitatory input driven by the contralateral ear is thought to arise from T-stellate or chopper cells which respond with sustained firing to most sustained sounds (Rhode et al., 1983) so cells receiving such synaptic input should exhibit sustained excitation to, for example, contralateral tones or noise. All three of our cell types showed such sustained excitatory inputs. For the uninhibited cells, this excitation was unaffected by any simultaneous inhibition and the cells fired for the duration of the stimulus (Figs. 8C, 10C, Suppl. Fig. 3). For the fast and slow cells sustained excitation was also observed and could generate repetitive firing but the firing was often reduced or eliminated by the sustained inhibition (Figs. 6B, 8A,B, 10A,B).

We also noted excitatory inputs driven from the ipsilateral ear. This was seen most clearly in the uninhibited cells (Suppl. Fig. 5) where it was unobstructed by ipsilateral inhibitory inputs. In these cells it was apparent that, like inhibition, the ipsilateral excitation was decidedly weaker than that activated by contralateral stimuli. Signs of ipsilateral excitatory inputs were also noted for fast and slow cells but these were weak and never able to exceed spike threshold. In the guinea pig a small percentage of the cochlear nucleus cells backfilled from SPN were ipsilateral (Schofield, 1995; Thompson and Thompson, 1991b) and their dendritic appearance and soma location led the authors to suggest they were octopus and multipolar (presumably T-stellate) cells so it is our assumption that these ipsilateral inputs also exist in the gerbil and may be responsible for the observed EPSPs.

A technical concern in the interpretation of laterality of sound-driven excitation or inhibition, particularly in the presence of threshold differences and in small animals, is the possibility of acoustic crosstalk, *i.e.* the spurious activation of one (“driving”) ear by stimuli presented to the other ear. This can occur through bone or air conduction or equipment supporting the animals and sound delivery systems. An additional reason for this concern was the opening of both bulla in our experiments: the ipsilateral bulla to gain access to the ventral brainstem and the contralateral bulla for reasons of acoustic symmetry. However, control experiments using the same preparation, reported elsewhere (Wei et al., 2017), suggest that the threshold difference between such direct and indirect effects are too large to account for the ipsilateral excitatory and inhibitory responses reported here.

### 4.6. Short tone responses

All previous reports of short tone responses arising from the SPN were obtained with extracellular recordings. These reports frequently classify the cell’s spike responses as onset, offset, a combination of on and off or some variety of sustained activity. Our data would indicate that some caution should be used as SPN cell responses to tones and other stimuli can vary depending on stimulus intensity and/or frequency. Fig. 8A shows a fast cell changing from onset to on/off to offset as the tone intensity increases. Fig. 10B shows a slow cell response to noise changing from sustained to offset as sound level increases.

### 4.7. Duration coding

In the rat, (Kadner et al., 2006) showed that as sound duration increased there was an increase in magnitude and decrease in latency of the offset spiking response generated by SPN neurons rebounding from inhibition implying that these cells are able to encode tone duration. In mouse (Kopp-Scheinpflug et al., 2011), this occurs because the strong, prolonged MNTB-generated inhibition activates an IH conductance more and more with increasing duration making the cell membrane “leakier” thus decreasing its time constant and generating a faster offset spike following offset of inhibition. At the same time the IPSP-induced hyperpolarization removes the inactivation of a low threshold calcium conductance resulting in more spikes generated upon repolarization from inhibition. If our supposition is correct that mice and rats only have fast SPN cells then these features are characteristic of this population. We did not test fast cells with tones of different durations, but we did such an experiment on a slow cell, giving a similar result (Fig. 11). This would indicate that the slow cell population may also use its MNTB inhibitory input to regulate these same conductances thus altering offset spike duration and number. Future slice experiments in gerbil or guinea pig can verify this possibility.

### 4.8. Gap detection

(Kadner and Berrebi, 2008) reported that SPN cells in rats are capable of detecting gaps as brief as 1 ms or less between tones. Gaps are an important cue in speech perception (Moore, 1993; Snell et al., 2002; Snell and Frisina, 2000). The proposed mechanism of gap detection in SPN neurons again incorporates the rebound spike(s) elicited by the offset of MNTB generated inhibition. This rebound spike occurs so rapidly that it is not influenced by the inhibition generated by the trailing tone. Again, our speculation is that in mice and rats the fast cells are the sole SPN cell type and that their fast membranes are capable of generating offset spikes with very short latencies (Fig. 5A). We did not test this in any of the fast cells but we did vary the duration of a gap between leading and trailing tones while recording from a slow cell. As Suppl. Fig. 4 illustrates it appears that the slower response times of the cell membrane for these cells makes them unsuited for detecting gaps of short duration as the onset of inhibition from the trailing tone suppresses the slow rebound spike.

### 4.9. Binaural interaction

SPN is not usually regarded as one of the superior olivary nuclei concerned with binaural cues (Felix and Magnusson, 2016; Magnusson and Gomez-Alvarez, 2019; Rajaram et al., 2020), but an SPN input from MSO, whose major function is extraction of ITDs, has been reported (Kuwabara and Zook, 1999). We provide evidence (Figs. 13 and 14) that these cells not only receive binaural inputs, but also show sensitivity to binaural cues important for sound localization. Although the binaural sensitivity is weak compared to that in the main binaural SOC nuclei (MSO, LSO), it is noteworthy that we found such sensitivity also in another periolivary location (Franken et al., 2016) using the same techniques. Thus, (weak) binaural sensitivity is perhaps more wide-spread than thought. Whether this sensitivity is pertinent to the targets of these inhibitory neurons remains to be determined.

## Supplementary Material

MMC1

## Data Availability

Data will be made available on request.

## References

[R1] Adams JC, 1981. Heavy metal intensification of DAB-based HRP reaction product. J. Histochem. Cytochem 29, 775. 7252134 10.1177/29.6.7252134

[R2] Banks MI, Smith PH, 1992. Intracellular recordings from neurobiotin-labeled cells in brain slices of the rat medial nucleus of the trapezoid body. J. Neurosci 12, 2819–2837. 1351938 10.1523/JNEUROSCI.12-07-02819.1992 PMC6575844

[R3] Behrend O, Brand A, Kapfer C, Grothe B, 2002. Auditory response properties in the superior paraolivary nucleus of the gerbil. J. Neurophysiol 87, 2915–2928. 12037195 10.1152/jn.2002.87.6.2915

[R4] Bremen P, Joris PX, 2013. Axonal recordings from medial superior olive neurons obtained from the lateral lemniscus of the chinchilla (Chinchilla laniger). J. Neurosci 33, 17506–17518. 10.1523/JNEUROSCI.1518-13.2013. 24174683 PMC6618368

[R5] Coleman JR, Clerici WJ, 1987. Sources of projections to subdivisions of the inferior colliculus in the rat. J. Comp. Neurol 262, 215–226. 3624552 10.1002/cne.902620204

[R6] Dehmel S, Kopp-Scheinpflug C, Dorrscheidt GJ, Rübsamen R, 2002. Electrophysiological characterization of the superior paraolivary nucleus in the Mongolian gerbil. Hear. Res 172, 18–36. 12361864 10.1016/s0378-5955(02)00353-2

[R7] Felix II RA, Gourevitch B, Gomez-Alvarez M, Leijon SCM, Saldaña E, Magnusson AK, 2017. Octopus cells in the posteroventral cochlear nucleus provide the main excitatory input to the superior paraolivary nucleus. Front. Neural Circ 11. 10.3389/fncir.2017.00037. PMC5449481 28620283

[R8] Felix RA 2nd, Fridberger A, Leijon S, Berrebi AS, Magnusson AK, 2011. Sound rhythms are encoded by postinhibitory rebound spiking in the superior paraolivary nucleus. J. Neurosci 31, 12566–12578. 10.1523/JNEUROSCI.2450-11.2011. 21880918 PMC3712520

[R9] Felix RA, Kadner A, Berrebi AS, 2012. Effects of ketamine on response properties of neurons in the superior paraolivary nucleus of the mouse. Neuroscience 201, 307–319. 10.1016/j.neuroscience.2011.11.027. 22123167 PMC3258328

[R10] Felix RA, Magnusson AK, 2016. Development of excitatory synaptic transmission to the superior paraolivary and lateral superior olivary nuclei optimizes differential decoding strategies. Neuroscience 334, 1–12. 10.1016/j.neuroscience.2016.07.039. 27476438

[R11] Felix RA, Vonderschen K, Berrebi AS, Magnusson AK, 2013. Development of on-off spiking in superior paraolivary nucleus neurons of the mouse. J. Neurophysiol 109, 2691–2704. 10.1152/jn.01041.2012. 23515791 PMC3680798

[R12] Franken TP, Bondy BJ, Haimes DB, Goldwyn JH, Golding NL, Smith PH, Joris PX, 2021. Glycinergic axonal inhibition subserves acute spatial sensitivity to sudden increases in sound intensity. Elife 10, e62183. 10.7554/eLife.62183. PMC8238506 34121662

[R13] Franken TP, Joris PX, Smith PH, 2018. Principal cells of the brainstem’s interaural sound level detector are temporal differentiators rather than integrators. Elife 7, e33854. 10.7554/eLife.33854. PMC6063729 29901438

[R14] Franken TP, Smith PH, Joris PX, 2016. In vivo whole-cell recordings combined with electron microscopy reveal unexpected morphological and physiological properties in the lateral nucleus of the trapezoid body in the auditory brainstem. Front. Neural Circ 10, 1–20. 10.3389/fncir.2016.00069. PMC4995217 27605909

[R15] Friauf E, Ostwald J, 1988. Divergent projections of physiologically characterized rat ventral cochlear nucleus neurons as shown by intra-axonal injection of horseradish peroxidase. Exp. Brain Res 73, 263–284. 3215304 10.1007/BF00248219

[R16] Gao F, Kadner A, Felix RA, Chen L, Berrebi AS, 2017. Forward masking in the superior paraolivary nucleus of the rat. Brain Struct. Funct 222, 365–379. 10.1007/s00429-016-1222-0. 27089883 PMC5069114

[R17] Godfrey DA, Kiang NYS, Norris BE, 1975. Single unit activity in the posteroventral cochlear nucleus of the cat. J. Comp. Neurol 162, 247–268. 1150921 10.1002/cne.901620206

[R18] Gómez-Álvarez M, Gourevitch B, Felix RA, Nyberg T, Hernández-Montiel HL, Magnusson AK, 2018. Temporal information in tones, broadband noise, and natural vocalizations is conveyed by differential spiking responses in the superior paraolivary nucleus. Eur. J. Neurosci 48, 2030–2049. 10.1111/ejn.14073. 30019495

[R19] Guinan JJ, Guinan SS, Norris BE, 1972a. Single auditory units in the superior olivary complex. I: responses to sounds and classifications based on physiological properties. Int. J. Neurosci 4, 101–120.

[R20] Guinan JJ, Norris BE, Guinan SS, 1972b. Single auditory units in the superior olivary complex. II: locations of unit categories and tonotopic organization. Int. J. Neurosci 4, 147–166.

[R21] Helfert RH, Bonneau JM, Wenthold RJ, Altschuler RA, 1989. GABA and glycine immunoreactivity in the guinea pig superior olivary complex. Brain Res. 501, 269–286. 2819441 10.1016/0006-8993(89)90644-6

[R22] Joris PX, Smith PH, 1998. Temporal and binaural properties in dorsal cochlear nucleus and its output tract. J. Neurosci 18, 10157–10170. 9822769 10.1523/JNEUROSCI.18-23-10157.1998 PMC6793293

[R23] Kadner A, Berrebi AS, 2008. Encoding of temporal features of auditory stimuli in the medial nucleus of the trapezoid body and superior paraolivary nucleus of the rat. Neuroscience 151, 868–887. 10.1016/j.neuroscience.2007.11.008. 18155850 PMC2267689

[R24] Kadner A, Kulesza RJ, Berrebi AS, 2006. Neurons in the medial nucleus of the trapezoid body and superior paraolivary nucleus of the rat may play a role in sound duration coding. J. Neurophysiol 95, 1499–1508. 10.1152/jn.00902.2005. 16319207

[R25] Kopp-Scheinpflug C, Pigott BM, Forsythe ID, 2015. Nitric oxide selectively suppresses IH currents mediated by HCN1-containing channels. J. Physiol 593, 1685–1700. 10.1113/jphysiol.2014.282194. 25605440 PMC4386966

[R26] Kopp-Scheinpflug C, Tozer AJB, Robinson SW, Tempel BL, Hennig MH, Forsythe ID, 2011. The sound of silence: ionic mechanisms encoding sound termination. Neuron 71, 911–925. 10.1016/j.neuron.2011.06.028. 21903083

[R27] Kulesza RJ Jr., Holt G, Spirou GA, Berrebi AS, 2000. Intracellular labeling of axonal collaterals of SPON neurons. Assoc. Res. Otolaryngol. Abs 23, 37.

[R28] Kulesza RJ, Berrebi AS, 2000. Superior paraolivary nucleus of the rat is a GABAergic nucleus. J. Assoc. Res. Otolaryngol 1, 255–269. 10.1007/s101620010054. 11547806 PMC2957197

[R29] Kulesza RJ Jr, 2007. Cytoarchitecture of the human superior olivary complex: medial and lateral superior olive. Hear. Res 225, 80–90. 10.1016/j.heares.2006.12.006. 17250984

[R30] Kulesza RJ, Kadner A, Berrebi AS, 2007. Distinct roles for glycine and GABA in shaping the response properties of neurons in the superior paraolivary nucleus of the rat. J. Neurophysiol 97, 1610–1620. 10.1152/jn.00613.2006. 17122321

[R31] Kulesza RJ, Spirou GA, Berrebi AS, 2003. Physiological response properties of neurons in the superior paraolivary nucleus of the rat. J. Neurophysiol 89, 2299–2312. 10.1152/jn.00547.2002. 12612016

[R32] Kulesza RJJ, Grothe B, 2015. Yes, there is a medial nucleus of the trapezoid body in humans. Front. Neuroanat 9. 10.3389/fnana.2015.00035. PMC4379933 25873865

[R33] Kuwabara N, DiCaprio RA, Zook JM, 1991. Afferents to the medial nucleus of the trapezoid body and their collateral projections. J. Comp. Neurol 314, 684–706. 1816271 10.1002/cne.903140405

[R34] Kuwabara N, Zook JM, 1999. Local collateral projections from the medial superior olive to the superior paraolivary nucleus in the gerbil. Brain Res. 846, 59–71. 10536214 10.1016/s0006-8993(99)01942-3

[R35] Kuwada S, Batra R, 1999. Coding of sound envelopes by inhibitory rebound in neurons of the superior olivary complex in the unanesthetized rabbit. J. Neurosci 19, 2273–2287. 10066278 10.1523/JNEUROSCI.19-06-02273.1999 PMC6782550

[R36] Leijon SCM, Peyda S, Magnusson AK, 2016. Temporal processing capacity in auditory-deprived superior paraolivary neurons is rescued by sequential plasticity during early development. Neuroscience 337, 315–330. 10.1016/j.neuroscience.2016.09.014. 27651152

[R37] Löhrke S, Srinivasan G, Oberhofer M, Doncheva E, Friauf E, 2005. Shift from depolarizing to hyperpolarizing glycine action occurs at different perinatal ages in superior olivary complex nuclei. Eur. J. Neurosci 22, 2708–2722. 10.1111/j.1460-9568.2005.04465.x. 16324105

[R38] Lu H-W, Smith PH, Joris PX, 2022. Mammalian octopus cells are direction selective to frequency sweeps by excitatory synaptic sequence detection. Proc. Natl. Acad. Sci. U. S. A 119, e2203748119. 10.1073/pnas.2203748119. PMC9636937 36279465

[R39] Lu H-W, Smith PH, Joris PX, 2018. Submillisecond monaural coincidence detection by octopus cells. Acta Acust. United Acust 104, 852–855. 10.3813/AAA.919238.

[R40] Magnusson AK, Gomez-Alvarez M, 2019. The superior paraolivary nucleus. In: Kandler K (Ed.), The Oxford Handbook of the Auditory Brainstem, Oxford Handbooks in Neuroscience. Oxford University Press, NY, pp. 395–419.

[R41] Mellott JG, Beebe NL, Schofield BR, 2018. GABAergic and non-GABAergic projections to the superior colliculus from the auditory brainstem. Brain Struct. Funct 223, 1923–1936. 10.1007/s00429-017-1599-4. 29302743 PMC5886796

[R42] Moore BCJ, 1993. Frequency Analysis and Pitch Perception, in: Human Psychophysics. Springer Handbook of Auditory Research Springer, New York, NY, pp. 56–115. 10.1007/978-1-4612-2728-1_3.

[R43] Morest DK, 1968. The collateral system of the medial nucleus of the trapezoid body of the cat, its neuronal architecture and relation to the olivo-cochlear bundle. Brain Res. 9, 288–311. 10.1016/0006-8993(68)90235-7. 5679830

[R44] Nayagam D, Clarey J, Paolini A, 2005. Powerful, onset inhibition in the ventral nucleus of the lateral lemniscus. J. Neurophysiol 94, 1651–1654. 15817650 10.1152/jn.00167.2005

[R45] Nordeen KW, Killackey HP, Kitzes LM, 1983. Ascending auditory projections to the inferior colliculus in the adult gerbil, Meriones unguiculatus. J. Comp. Neurol 214, 131–143. 10.1002/cne.902140203. 6841681

[R46] Oertel D, Bal R, Gardner SM, Smith PH, Joris PX, 2000. Detection of synchrony in the activity of auditory nerve fibers by octopus cells of the mammalian cochlear nucleus. Proc. Natl. Acad. Sci. U. S. A 97, 11773–11779. 10.1073/pnas.97.22.1177397/22/11773 [pii]. 11050208 PMC34348

[R47] Ostapoff EM, Benson CG, Saint Marie RL, 1997. GABA- and glycine-immunoreactive projections from the superior olivary complex to the cochlear nucleus in guinea pig. J. Comp. Neurol 381, 500–512. 10.1002/(sici)1096-9861(19970519)381, 4<500::aid—cne9>3.0.co;2-6. 9136806

[R48] Pecka M, Brand A, Behrend O, Grothe B, 2008. Interaural time difference processing in the mammalian medial superior olive: the role of glycinergic inhibition. J. Neurosci 28, 6914–6925. 18596166 10.1523/JNEUROSCI.1660-08.2008 PMC6670983

[R49] Radtke-Schuller S, Schuller G, Angenstein F, Grosser OS, Goldschmidt J, Budinger E, 2016. Brain atlas of the Mongolian gerbil (Meriones unguiculatus) in CT/MRI-aided stereotaxic coordinates. Brain Struct. Funct 221, 1–272. 10.1007/s00429-016-1259-0. PMC5005445 27507296

[R50] Rajaram E, Kaltenbach C, Fischl MJ, Mrowka L, Alexandrova O, Grothe B, Hennig MH, Kopp-Scheinpflug C, 2019. Slow NMDA-mediated excitation accelerates offset-response latencies generated via a post-inhibitory rebound mechanism. eNeuro 6. 10.1523/ENEURO.0106-19.2019. ENEURO.0106–19.2019. PMC6584069 31152098

[R51] Rajaram E, Pagella S, Grothe B, Kopp-Scheinpflug C, 2020. Physiological and anatomical development of glycinergic inhibition in the mouse superior paraolivary nucleus following hearing onset. J. Neurophysiol 124, 471–483. 10.1152/jn.00053.2020. 32667247

[R52] Rhode WS, Smith PH, 1986. Physiological studies on neurons in the dorsal cochlear nucleus of cat. J. Neurophysiol 56, 287–307. 3760922 10.1152/jn.1986.56.2.287

[R53] Rhode WS, Smith PH, Oertel D, 1983. Physiological response properties of cells labeled intracellularly with horseradish peroxidase in cat dorsal cochlear nucleus. J. Comp. Neurol 213, 426–447. 10.1002/cne.902130407. 6300199

[R54] Roberts MT, Seeman SC, Golding NL, 2014. The relative contributions of MNTB and LNTB neurons to inhibition in the medial superior olive assessed through single and paired recordings. Front. Neural Circ 8, 49. 10.3389/fncir.2014.00049. PMC4030206 24860434

[R55] Roberts MT, Seeman SC, Golding NL, 2013. A mechanistic understanding of the role of feedforward inhibition in the mammalian sound localization circuitry. Neuron 78, 923–935. 10.1016/j.neuron.2013.04.022. 23764291 PMC3690812

[R56] Saldaña E, Aparicio M-A, Fuentes-Santamaría V, Berrebi AS, 2009. Connections of the superior paraolivary nucleus of the rat: projections to the inferior colliculus. Neuroscience 163, 372–387. 10.1016/j.neuroscience.2009.06.030. 19539725 PMC2778228

[R57] Saldaña E, Berrebi AS, 2000. Anisotropic organization of the rat superior paraolivary nucleus. Anat. Embryol. (Berl.) 202, 265–279. 10.1007/s004290000109. 11000278

[R58] Schofield BR, 1995. Projections from the cochlear nucleus to the superior paraolivary nucleus in guinea pigs. J. Comp. Neurol 360, 135–149. 10.1002/cne.903600110. 7499559

[R59] Schofield BR, 1991. Superior paraolivary nucleus in the pigmented guinea pig: separate classes of neurons project to the inferior colliculus and the cochlear nucleus. J. Comp. Neurol 312, 68–76. 10.1002/cne.903120106. 1744244

[R60] Schofield BR, Cant NB, 1992. Organization of the superior olivary complex in the guinea pig: II. Patterns of projection from the periolivary nuclei to the inferior colliculus. J. Comp. Neurol 317, 438–455. 10.1002/cne.903170409. 1578006

[R61] Schofield BR, Cant NB, 1991. Organization of the superior olivary complex in the guinea pig. I. Cytoarchitecture, cytochrome oxidase histochemistry, and dendritic morphology. J. Comp. Neurol 314, 645–670. 1726174 10.1002/cne.903140403

[R62] Smith PH, Joris PX, Banks MI, Yin TCT, 1993. Responses of cochlear nucleus neurons and projections of their axons, in: The Mammalian Cochlear Nuclei: Organization and Function. Plenum, New York, pp. 349–360.

[R63] Smith PH, Joris PX, Carney LH, Yin TC, 1991. Projections of physiologically characterized globular bushy cell axons from the cochlear nucleus of the cat. J. Comp. Neurol 304, 387–407. 10.1002/cne.903040305. 2022755

[R64] Smith PH, Joris PX, Yin TC, 1998. Anatomy and physiology of principal cells of the medial nucleus of the trapezoid body (MNTB) of the cat. J. Neurophysiol 79, 3127–3142. 9636113 10.1152/jn.1998.79.6.3127

[R65] Smith PH, Massie A, Joris PX, 2005. Acoustic stria: anatomy of physiologically characterized cells and their axonal projection patterns. J. Comp. Neurol 482, 349–371. 10.1002/cne.20407. 15669051

[R66] Snell KB, Frisina DR, 2000. Relationships among age-related differences in gap detection and word recognition. J. Acoust. Soc. Am 107, 1615–1626. 10.1121/1.428446. 10738815

[R67] Snell KB, Mapes FM, Hickman ED, Frisina DR, 2002. Word recognition in competing babble and the effects of age, temporal processing, and absolute sensitivity. J. Acoust. Soc. Am 112, 720–727. 10.1121/1.1487841. 12186051

[R68] Sommer I, Lingenhohl K, Friauf E, 1993. Principal cells of the rat medial nucleus of the trapezoid body: an intracellular in vivo study of their physiology and morphology. Exp. Brain Res 95, 223–239. 8224048 10.1007/BF00229781

[R69] Spangler KM, Cant NB, Henkel CK, Farley GR, Warr WB, 1987. Descending projections from the superior olivary complex to the cochlear nucleus of the cat. J. Comp. Neurol 259, 452–465. 3584567 10.1002/cne.902590311

[R70] Thompson AM, Thompson GC, 1991a. Projections from the posteroventral cochlear nucleus to the superior olivary complex in guinea pig: light and EM observations with the PHA-L method. J. Comp. Neurol 311, 495–508. 10.1002/cne.903110405. 1757599

[R71] Thompson AM, Thompson GC, 1991b. Posteroventral cochlear nucleus projections to olivocochlear neurons. J. Comp. Neurol 303, 267–285. 10.1002/cne.903030209. 2013640

[R72] Walton JP, 2010. Timing is everything: temporal processing deficits in the aged auditory brainstem. Hear. Res 264, 63–69. 10.1016/j.heares.2010.03.002. 20303402 PMC7045868

[R73] Warr WB, 1972. Fiber degeneration following lesions in the multipolar and globular cell areas in the ventral cochlear nucleus of the cat. Brain Res. 40, 247–270. 10.1016/0006-8993(72)90132-1. 5027165

[R74] Wei L, Karino S, Verschooten E, Joris PX, 2017. Enhancement of phase-locking in rodents. I. An axonal recording study in gerbil. J. Neurophysiol 118, 2009–2023. 10.1152/jn.00194.2016. 28701535 PMC5626893

[R75] Willard FH, Ryugo DK, 1983. Anatomy of the central auditory system, in: WIllott JF, The Auditory Psychobiology of the Mouse. Charles C. Thomas, Springfield, IL, pp. 201–304.

[R76] Yassin L, Radtke-Schuller S, Asraf H, Grothe B, Hershfinkel M, Forsythe ID, Kopp-Scheinpflug C, 2014. Nitric oxide signaling modulates synaptic inhibition in the superior paraolivary nucleus (SPN) via cGMP-dependent suppression of KCC2. Front. Neural Circ 8, 65. 10.3389/fncir.2014.00065. PMC4060731 24987336

[R77] Zook JM, Casseday JH, 1985. Projections from the cochlear nuclei in the mustache bat, Pteronotus parnellii. J. Comp. Neurol 237, 307–324. 10.1002/cne.902370303. 2995459

