# Supplementary material for: Distinct cell classes in the superior paraolivary nucleus (SPN) region in the gerbil auditory brainstem revealed by in vivo physiological and anatomical characterization
Source: Hear Res. Author manuscript; Available in PMC 2025 Apr 24. (PMC12019997; doi:10.1016/j.heares.2025.109202)
Supplement: MMC1 [file NIHMS2058256-supplement-MMC1.docx]

**SUPPLEMENTARY FIGURES**

**
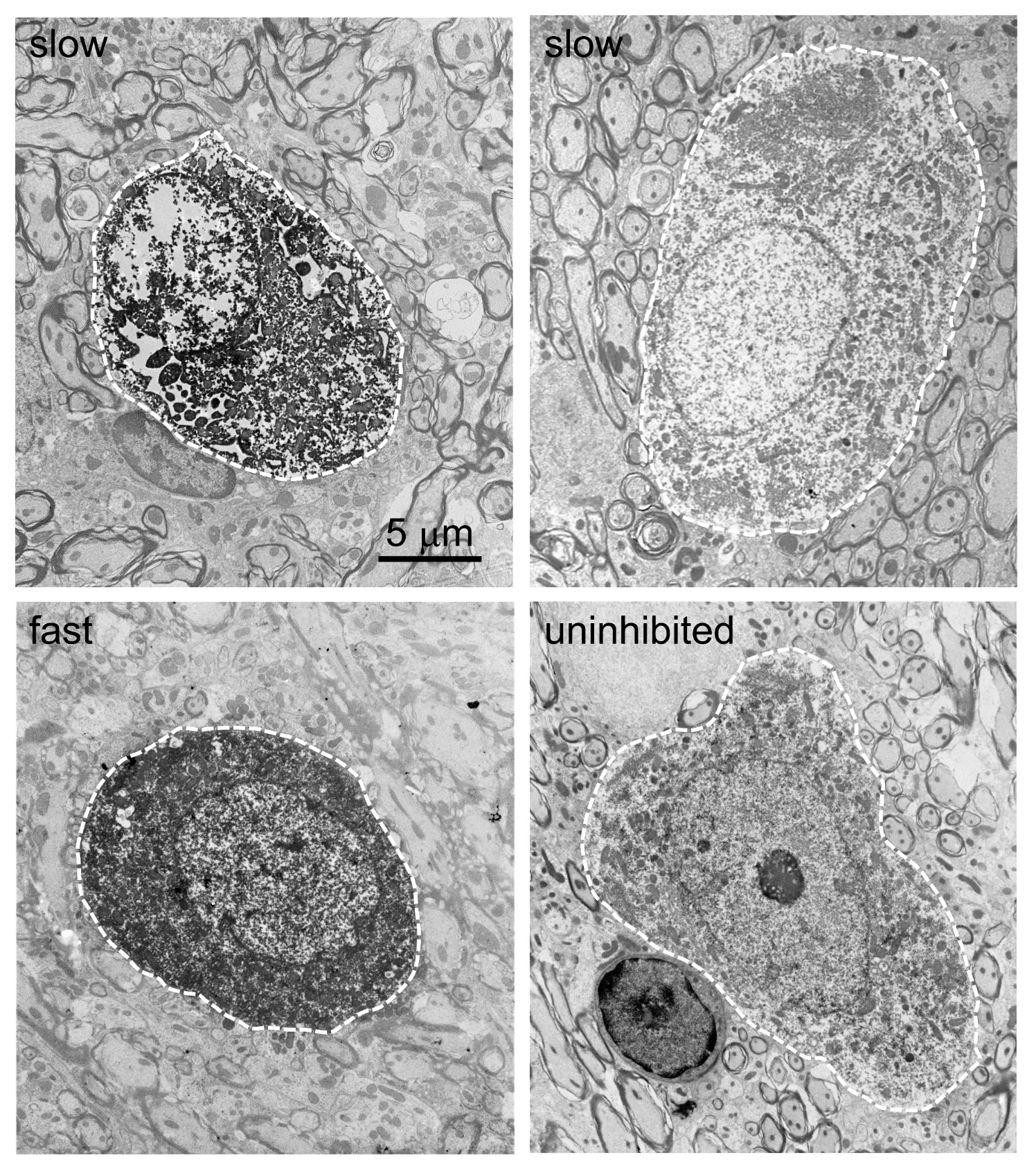
**

**Suppl. Fig. 1.** Electron micrographs of 2 slow (top) 1 fast (bottom left) and 1 uninhibited (bottom right) SPN cell bodies are outlined with dotted lines for clarity. Scale bar applies to all micrographs.

**
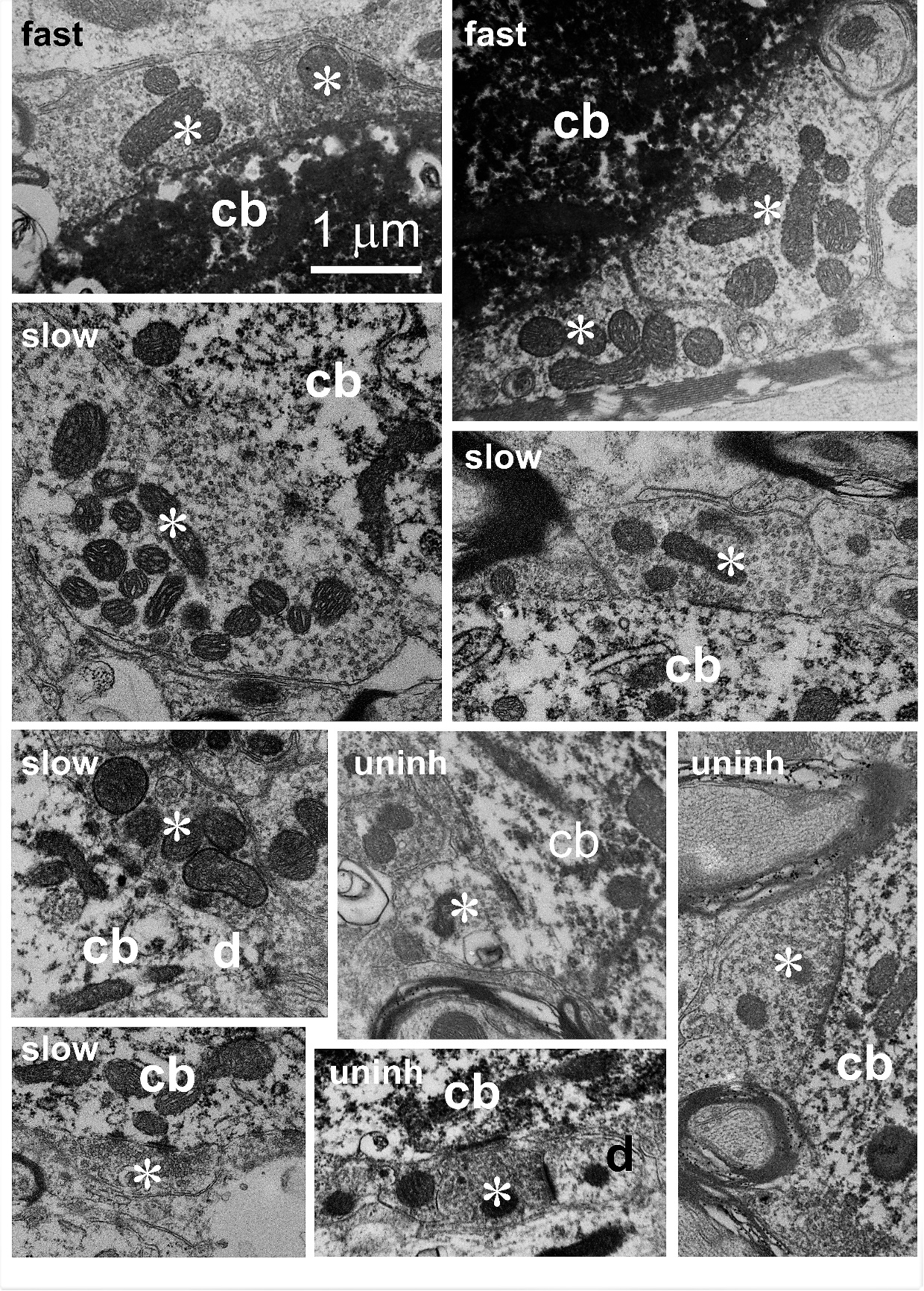
**

**Suppl. Fig. 2**. Examples of synaptic terminals (asterisks) on labeled fast, slow and uninhibited SPN cell bodies (cb). In the second “slow” terminal on the left the synapse is at the intersection of the cell body and a dendrite (d). In the lower middle micrograph, the terminal is synapsing on a labeled cell body as well as an unlabeled dendrite (d). Scale bar in top left micrograph applies to all micrographs.

**
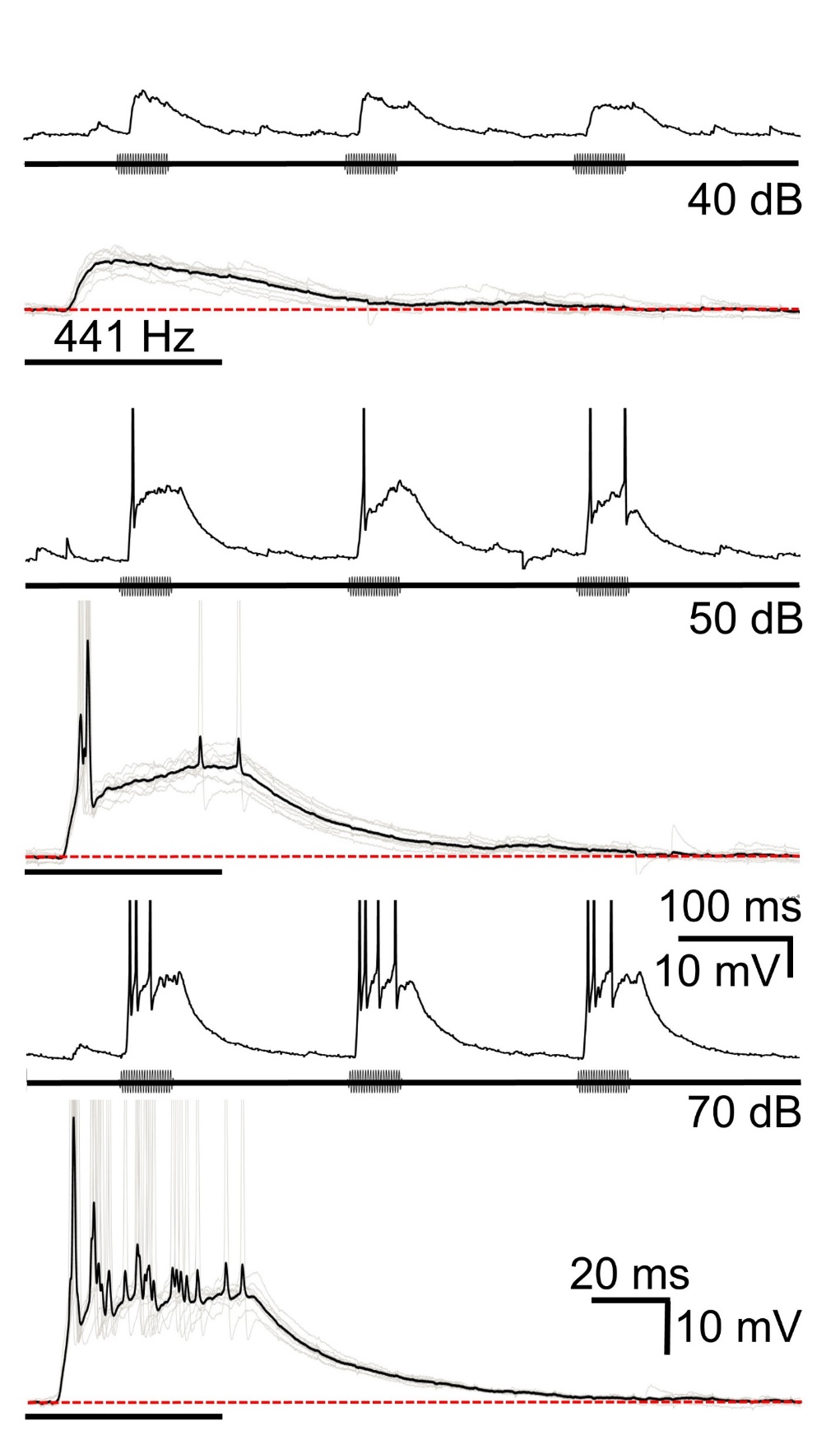
**

**Suppl. Fig. 3.** Averaged responses of uninhibited cell to CF tones at different sound levels. 1st, 3rd and 5th traces are responses to 3 tones of 50 ms at 441 Hz. 2nd, 4th and 6th traces are averaged responses to a single 50 ms tone. Dotted red line = resting potential. Upper (100-ms) scale bar applies to traces 1,3,5. Lower (20-ms) scale bar applies to traces 2,4,6. Spikes are reduced due to averaging.

**
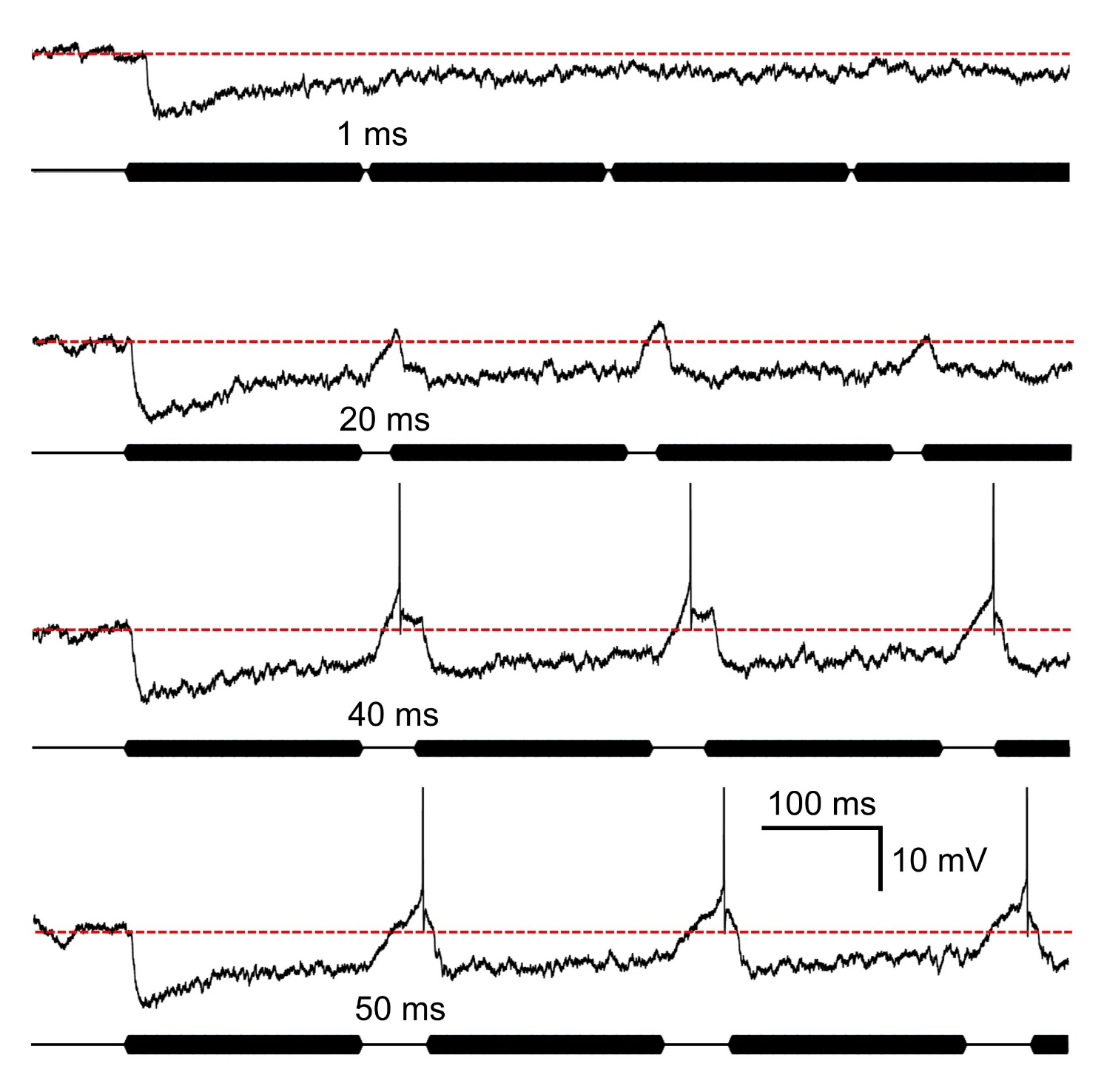
**

**Suppl. Fig. 4.** Response of a slow cell to 200-ms tones at different gap durations. At intervals of 40 and 50 ms (lower 2 traces) the rebound from inhibition is able to generate an action potential indicating the presence of a gap. At shorter intervals below 40 ms the slow return to baseline of the inhibition is interrupted by the next tone-generated inhibitory response suppressing the spike. Scale bars near bottom trace applies to all traces. Dotted red dotted line = resting membrane potential. Spikes are clipped to fit.

**
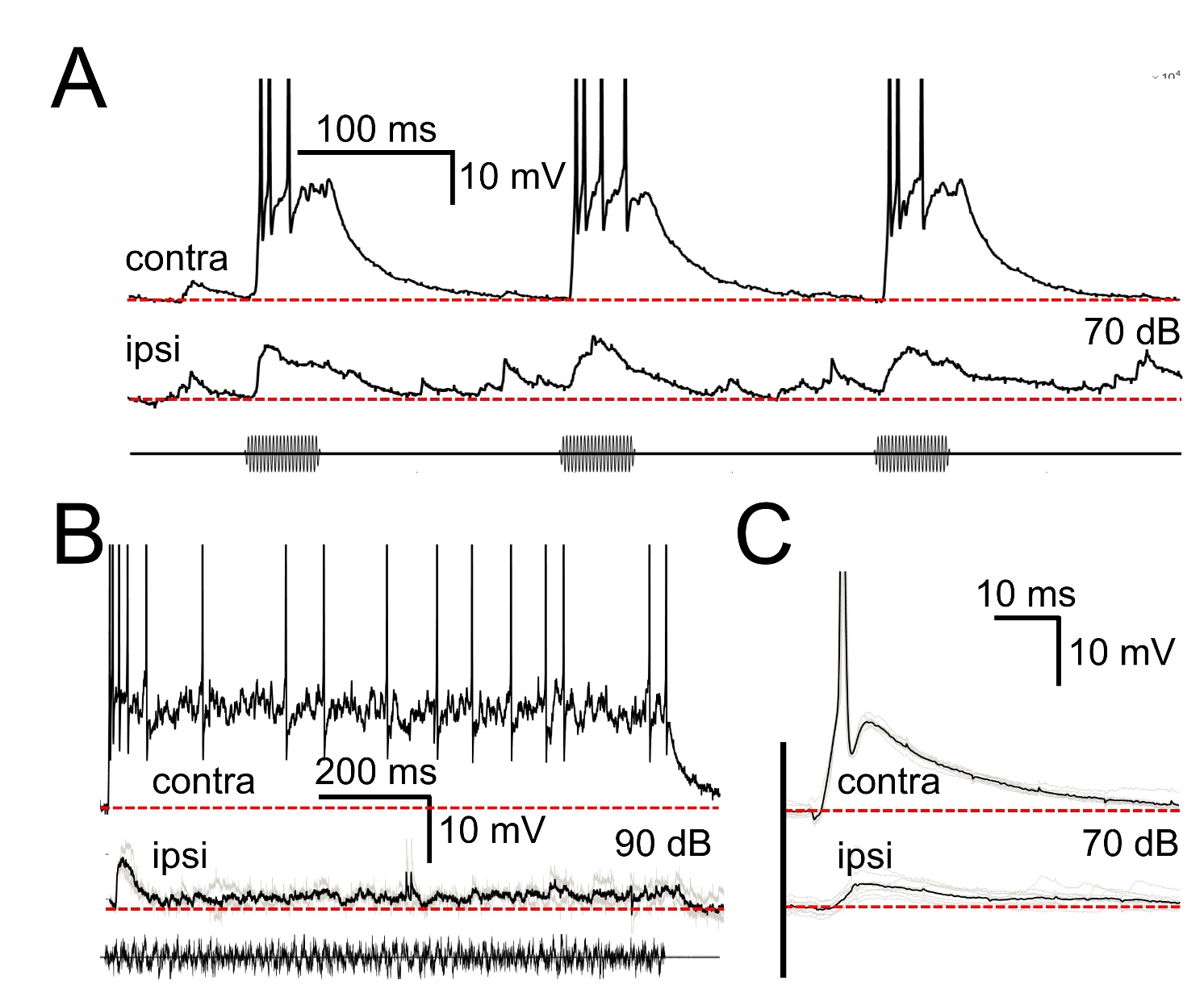
**

**Suppl. Fig. 5.** Comparison of the responses of an uninhibited cell to contralateral and ipsilateral stimuli. A. Response to 3 contralateral or ipsilateral tones (441 Hz, 70 dB, 50 ms). B. Response to a contralateral or ipsilateral 1-s noise burst at 90 dB. C. Response to a contralateral and ipsilateral click at 70 dB. Red dotted line = resting potential. Spikes are clipped to fit.
